# On the potential of drug repurposing in dysphagia treatment: New insights from a real-world pharmacovigilance study and a systematic review

**DOI:** 10.3389/fphar.2023.1057301

**Published:** 2023-03-03

**Authors:** Vera Battini, Sara Rocca, Greta Guarnieri, Anna Bombelli, Michele Gringeri, Giulia Mosini, Marco Pozzi, Maria Nobile, Sonia Radice, Emilio Clementi, Antonio Schindler, Carla Carnovale, Nicole Pizzorni

**Affiliations:** ^1^ Department of Biomedical and Clinical Sciences, Pharmacovigilance & Clinical Research, International Centre for Pesticides and Health Risk Prevention, “Luigi Sacco” University Hospital, Università degli Studi di Milano, Milan, Italy; ^2^ Phoniatric Unit, Department of Biomedical and Clinical Sciences, “Luigi Sacco” University Hospital, Università degli Studi di Milano, Milan, Italy; ^3^ Scientific Institute IRCCS Eugenio Medea, Bosisio Parini(LC), Italy

**Keywords:** substance P, deglutition disorders, dipeptidyl-peptidase IV inhibitors, adrenergic β-antagonists, angiotensin-converting enzyme inhibitors

## Abstract

**Background:** Polypharmacy is common in patients with dysphagia. Routinely used drugs may influence swallowing function either improving or worsening it. We aimed to explore the potential effects of three commonly used drug classes on dysphagia and aspiration pneumonia through a systematic review and a real-world data analysis to probe the possibility of drug repurposing for dysphagia treatment.

**Material and Methods:** Five electronic databases were searched. Studies on adults at risk for dysphagia, treated with Dipeptidyl-Peptidase IV Inhibitors (DPP-4i), Adrenergic Beta-Antagonists (beta-blockers), or Angiotensin-Converting Enzyme Inhibitors (ACEi), and reporting outcomes on dysphagia or aspiration pneumonia were included. A nested case/non-case study was performed on adverse events recorded in the FDA Adverse Event Reporting System (FAERS) on patients >64 years. Cases (dysphagia or aspiration pneumonia) were compared between patients only treated with Levodopa and patients who were concomitantly treated with the drugs of interest.

**Results:** Twenty studies were included in the review (17 on ACEi, 2 on beta-blockers, and 1 on DPP-4i). Contrasting findings on the effects of ACEi were found, with a protective effect mainly reported in Asian studies on neurological patients. Beta-blockers were associated with a reduced dysphagia rate. The study on DPP-4i suggested no effect on dysphagia and an increased risk of aspiration pneumonia. The FAERS analysis showed a reduction of the risk for dysphagia/aspiration pneumonia with ACEi, beta-blockers, and DPP-4i.

**Conclusion:** Our study explores the potential drug repurposing of ACEi, beta-blockers and DPP-4i in neurological patients with dysphagia to improve swallowing function and reduce aspiration pneumonia risk. Future randomized controlled studies should confirm these results and clarify the underlying mechanisms of action.

## 1 Introduction

Dysphagia is an impairment in the bolus transit from the mouth to the stomach (Merlo and Cohen, 1988). It may result from a variety of conditions such as neurological diseases, head and neck cancer, chronic respiratory disease, and aging. Its prevalence in the general population is 12.1% (Kertscher et al., 2015), but it dramatically increases in high-risk populations such as patients with stroke (up to 80%), Parkinson’s disease (up to 81%), and community-acquired pneumonia (91.7%) (Takizawa et al., 2016). Polypharmacy is therefore common in patients with dysphagia due to the symptoms that are associated with the underlying disease and the increasing number of comorbidities while aging, such as hypertension and diabetes mellitus (Miarons et al., 2016; Wolf et al., 2021). Unfortunately, detrimental effects on swallowing function have been reported for several drug classes commonly prescribed to the elderly patients, such as antidepressants, antipsychotics, benzodiazepines, antiepileptics, and drugs for dementia (Miarons et al., 2016; Dzahini et al., 2018; Wolf et al., 2021), thus exposing patients with dysphagia to an additional risk for pulmonary and nutritional complications.

Indeed, dysphagia is associated with severe complications, such as aspiration pneumonia—the leading cause of death in many neurodegenerative diseases (Lanska et al., 1988; Auyeung et al., 2012; Heemskerk and Roos, 2012), malnutrition, and dehydration: these conditions impact survival, clinical management, and health costs (Attrill et al., 2018; Marin et al., 2021). Thus, preventing dysphagia-related complications by early identification and treatment of dysphagia is of crucial importance. Mechanisms associated with the worsening of dysphagia are various and include xerostomia, drug-induced extrapyramidal symptoms, interactions with neural pathways involved in swallowing, and medicinal injury to the mucous membranes of the structures involved in swallowing. Conversely, potential beneficial effects on dysphagia have been suggested for some pharmacological agents. ACEi and Dipeptidyl-Peptidase IV Inhibitors (DPP-4i) have been reported to improve the swallowing reflex (Cunningham and O'Connor, 1997; Nakayama et al., 1998). Beta-blockers were found to be associated with lower dysphagia prevalence in the elderly patients (Miarons et al., 2018). Nevertheless, none of the studies clarified the mechanisms underlying the potential positive effects of these drugs on dysphagia, although a weak role of substance P (SP), a neuropeptide that enhances swallowing and cough reflexes, was hypothesized (Jin et al., 1994; Imoto et al., 2011; Canning et al., 2014). As many drugs have pleiotropic effects because of their interaction with multiple biological targets, known as primary and secondary effects (Jourdan et al., 2020; Hua et al., 2022), the process of finding new uses outside the original approved medical indication for existing drugs—i.e., redirecting, repurposing, repositioning and reprofiling (Kerber, 2003; Longman, 2004; Stuart, 2004), is increasing attention.

At present, dysphagia treatment relies on a variety of approaches including behavioral treatment, alternative feeding methods, neurostimulation techniques, surgical approaches, and pharmacological treatment. However, the pharmacological approach to dysphagia is relatively recent and still poorly investigated: a recent systematic review on the topic concluded that the number of randomized controlled trials (RCTs) for most of the pharmacological agents is very limited and the evidence of their efficacy is still scant (Cheng et al., 2022).

As a consequence of the lack of clinical trials, despite the intrinsic limitations, the use of alternative source (including pharmacovigilance databases) for retrieving potential effective additional uses of drugs has increased exponentially (Gatti et al., 2021; Ganesh and Randall, 2022).

The aim of this study is then to explore the potential effects of some drug classes on dysphagia and aspiration pneumonia through a systematic review and a real-world data analysis from the US Food and Drug Administration Adverse Event Reporting System (FAERS) database. In particular, we focused on three drug classes routinely used in clinical practice: DPP-4i, beta-blockers, and ACEi. These drug classes were selected because they are frequently prescribed in patients with dysphagia (Miarons et al., 2016; Wolf et al., 2021) and share a common secondary target, the aforementioned cleavage of SP. On the other hand, to target a population at high risk of dysphagia (Baijens et al., 2016; Takizawa et al., 2016), the real-data analysis was focused on the reports of patients aged >64 years and treated with Levodopa (i.e., subjects with Parkinson’s disease). The results of this exploratory study may help generate new hypotheses on the potential of drug repurposing for dysphagia treatment, to be verified in future randomized controlled trials.

## 2 Methods

### 2.1 Systematic review

#### 2.1.1 Search strategy

We performed a systematic review in accordance with the Preferred Reporting Items for Systematic Reviews and Meta-Analyses (PRISMA) guidelines (Page et al., 2021). We searched PubMed, Embase, CINHAL, Scopus, and the Cochrane electronic databases from inception up to 14 August 2021 with no language restriction. Our search strategy was adapted as necessary for each database and complete details of each search are described in Supplementary Table S1. Text words and database subject headings were used that were synonymous with the interventions and the outcomes of interest. Essentially, we used the following search terms.

- Intervention: DPP-4i, Beta-blockers, ACEi, Neprilysin inhibitors (ACNi);
- Outcome: dysphagia, aspiration pneumonia.

The terms related to the intervention and the outcome were combined with the Boolean operator “AND”. Additionally, the reference lists of the included studies and relevant reviews were checked for other potentially relevant studies. As no results for ACNi were found, this drug class will not be mentioned in the results and discussion sections.

#### 2.1.2 Eligibility criteria

Study eligibility was based on inclusion and exclusion criteria regarding population, intervention, outcome, study design, and publication type. More specifically, inclusion criteria were: (i) studies on adult patients with any condition commonly associated with the onset of dysphagia (e.g., neurodegenerative diseases, stroke, head and neck diseases, and geriatric patients); (ii) studies including patients treated with any DPP-4i, Beta-blockers, ACEi or ACNi; (iii) studies reporting outcomes on swallowing function, aspiration pneumonia or SP concentrations; (iv) randomized (RCT), non-randomized clinical trials (nRCT), single-arm clinical trials (CTs), and observational studies. Literature reviews, case reports, and unpublished thesis were excluded.

#### 2.1.3 Study selection

The records identified from the electronic search were imported into the software Rayyan (Ouzzani et al., 2016). After duplicate removal, our search results were screened by title and abstract for potentially eligible studies by two independent researchers (NP and VB). Potentially relevant studies were retrieved in full text and assessed for eligibility based on our prespecified inclusion criteria by two independent researchers (AB and SR). Reasons for the exclusion of full texts were recorded. Disagreements about eligibility were resolved by consensus.

#### 2.1.4 Data extraction

Data from all included studies were extracted by two independent researchers (AB and SR) using pre-specified forms. Disagreements were resolved by consensus and consultation with the expert group (NP and VB). For each included study, the following information was extracted: first author, year of publication, study design, study duration, number of subjects, patient diagnoses, age (mean, median, range, standard deviation, interquartile range), sex distribution, generic name of the drug of interest, drug dose, concomitant therapies (concomitant drugs or swallowing rehabilitation), outcomes of interest reported in the study, assessment method for dysphagia, percentage of patients with dysphagia at the baseline and post-treatment, definition of aspiration pneumonia, percentage of patients with first event of recurrence of aspiration pneumonia, serum SP concentrations at the baseline and post-treatment, and main results. We did not contact authors for missing data.

#### 2.1.5 Quality and risk of bias

Two authors (SR and GG) assessed the risk of bias of RCTs by using the Cochrane risk-of-bias tool for randomized trials (RoB2) (Sterne et al., 2019) and of nRCTs, single-arm CTs, and observational studies by using the risk of bias tool to assess non-randomized studies of interventions (ROBINS-I tool) (Sterne et al., 2016). For single-arm studies, a modified version of the ROBINS-I tool was used. Disagreements were resolved by consensus and consultation with the expert group (NP and VB).

### 2.2 Pharmacovigilance study

#### 2.2.1 Data source and extraction

Data were obtained from the FAERS, one of the largest and most comprehensive spontaneous reporting system databases. It contains information related to post-marketing safety surveillance reports in the form of adverse events (AEs) submitted by healthcare professionals, consumers, and other sources. AEs are recorded in the FAERS using the Medical Dictionary for Regulatory Activities (MedDRA^®^) preferred terms (PTs) (Fescharek et al., 2004), as Individual Case Safety Reports (ICSRs). Each ICSR provides administrative information (country, type of report, qualification of the reporter), patient demographics (sex, age, weight), AE characteristics (seriousness, date of onset, outcome), details about suspect drug therapy (drug name, exposure start and stop dates, time to onset, dose, route, indication, de-challenge and re-challenge) and information concerning any drug administered at the time of AE but not held responsible for its occurrence by the reporter, referred to as concomitant medication. However, the level of completeness of information varies from case to case (Sakaeda et al., 2013).

As the number of safety reports sent to the FDA annually is continuously growing, the database is largely used to detect novel drug-related safety events, to identify possible mechanisms of adverse events, to explore potential drug-drug interactions related to adverse events, and to discover promising new concomitant uses of drugs (Carnovale et al., 2019a; Mazhar et al., 2019; Mazhar et al., 2021).

Adverse events recorded in the FAERS were downloaded from the Food and Drug Administration (FDA) website (US FDA FAERS). The database consists of seven datasets, namely, patient demographic and administrative information (file descriptor DEMO), drug and biologic information (DRUG), adverse events (REAC), patient outcomes (OUTC), report sources (RPSR), start and end dates of drug therapy (THER), and indications for use/diagnosis (INDI). These seven datasets were joined by unique identification numbers for each FAERS report and a relational database was built. Data extraction was restricted to reports without missing values for age and gender; when more versions of the same ICSR were available, the last one was retained. Duplicate records were automatically detected and deleted by comparing the following information among the ICSRs: age, sex, event date, primary suspect, and country. Names of pharmaceutical drugs were harmonized by using the American RxTerms terminology (National Library of Medicine, 2020). The final cleaning process removed the list of “deleted cases” provided by the FDA and cases reported from the literature (US FDA FAERS).

This study was designed as a nested case/non-case study. The cohort was retrieved from the FAERS database in the period covering the first quarter of 2010 to the third quarter of 2021 and consisted of reports involving patients with more than 64 years (Baijens et al., 2016); in general, this population is known to be at risk for swallowing difficulties. Since the use and approval of drugs varies significantly between countries, we limited data extraction to Individual Case Safety Reports (ICSRs) from North America and Europe (except eastern countries). After a review of all LLTs in MedDRA (Fescharek et al., 2004), two terms were selected as relevant descriptors of the ADR of interest: Dysphagia and “Pneumonia Aspiration”. ICSRs reporting at least one of the LLT above mentioned were considered “cases” whilst “non-cases” were all the other ICSRs reporting other AEs.

#### 2.2.2 Statistical analysis

Descriptive analysis was performed in terms of age, female sex, reporter type, country and the use of concomitant medications known to increase the risk of dysphagia (the list was retrieved from ClinicalKey (Brown, 2013; Clinical Pharmacology, 2020) and from Miarons et al. (2016); see Supplementary Table S2). Between-group differences for the continuous variables were analyzed by the Mann-Whitney *U* test while categorical variables (sex, country, and the presence of concomitant medications) analyzed by Pearson’s Chi-square test. Tests were two-tailed, with significance set at a *p*-value of 0.05.

The crude (cROR) and adjusted reporting odds ratio (aROR) were calculated using univariate and multivariate logistic regression analysis respectively, and adjusted for potential confounding factors such as age class, gender, and concomitant drugs that are known to increase the risk of dysphagia. Since it is known that, among people aged over 64, patients with Parkinson’s are at a high risk of developing dysphagia (Chaudhuri et al., 2006; Takizawa et al., 2016; Wolf et al., 2021; Wang et al., 2022), we compared cases between those who were treated with Levodopa only and those who were concomitantly treated at least with a drug inhibiting the degradation of the SP: ACEi [WHO Anatomical Therapeutic Chemical (ATC) code: C09A]; beta-blockers [ATC code: C07A], Gliptins [ATC code: A10BH], ACNi (sacubitril) [ATC code: C09DX]. The reference group consisted of ICSRs where none of the above-mentioned drugs were reported. We assumed that reports involving only Levodopa would have an increased reporting risk compared to the general population aged over 64 and that the concomitant use of beta-blockers/ACEi/DPP-4i would reduce that risk. Signals of disproportionate reporting were detected when the number of reports was ≥3 and ROR—95% CI was greater than one.

Finally, since dysphagia is a condition that requires a specific clinical diagnosis, we planned a sensitivity analysis by using only ICSRs reported by physicians, in order to control for the potential confounding of this covariate. All analyses were performed using counts of unique cases.

Data reading, filtering, processing, and statistical analysis were performed through RStudio.

## 3 Results

### 3.1 Systematic review

#### 3.1.1 Search process

**FIGURE 1:**
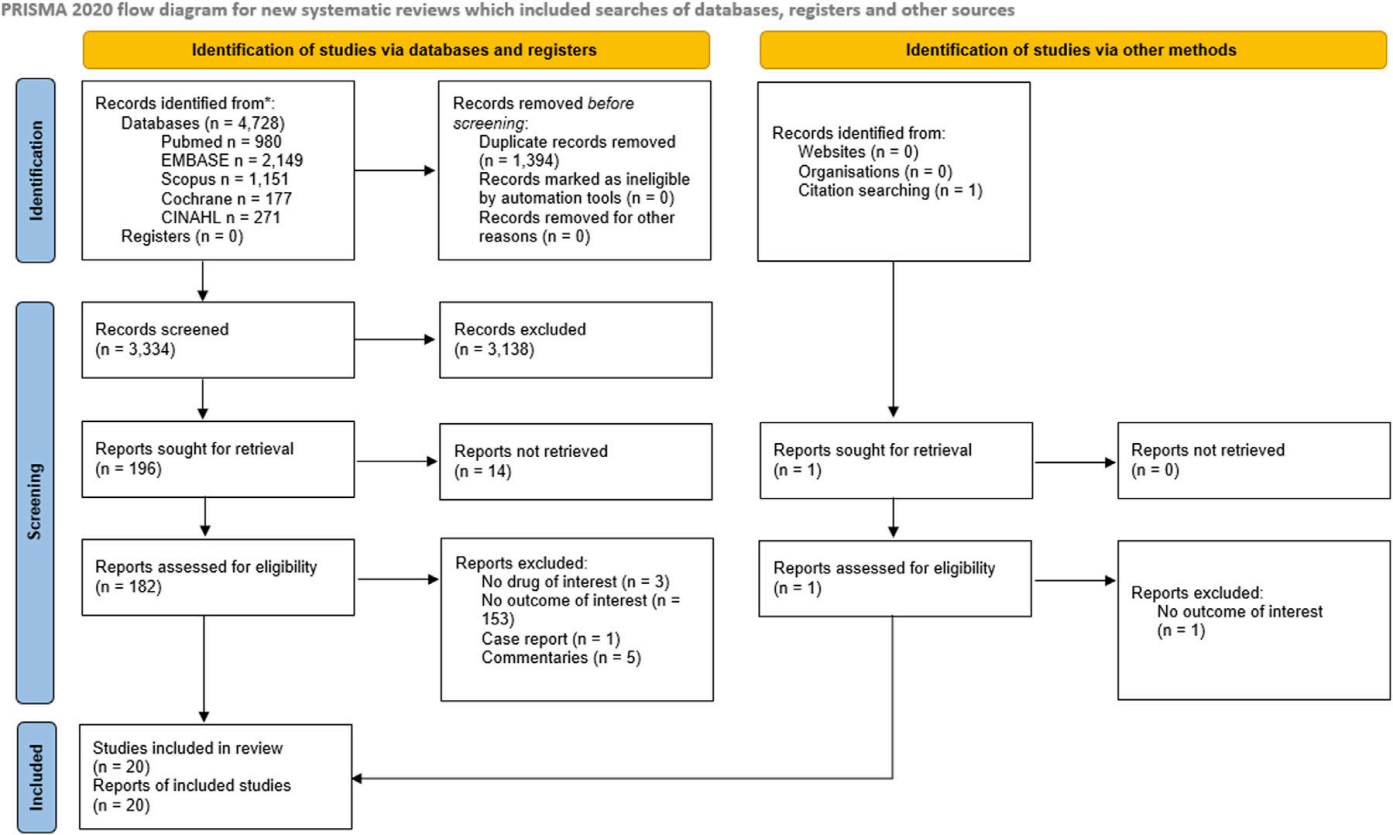
PRISMA 2020 flow diagram depicting the flow of information through the different phases of the Systematic review.

The study selection and screening process is presented in the PRISMA flowchart (Figure 1). The electronic search identified 4,728 records. After duplicate removal, 3,334 records were screened. One record was retrieved by manual search in the reference lists of relevant reviews and included studies for full-text analysis. In total, 183 full-text articles were assessed for eligibility. Ultimately, 20 studies met the eligibility criteria and were included in the review (Table 1). The effects of ACEi (Arai et al., 1998a; Arai et al., 1998b; Nakayama et al., 1998; Arai et al., 2001; Arai et al., 2003; Arai et al., 2005; Shimizu et al., 2008; Marciniak et al., 2009; Nakashima et al., 2011; Bosch et al., 2012; Liu et al., 2012; Matsumoto et al., 2012; Lee et al., 2015; Kano et al., 2016; Kumazawa et al., 2019; Fernandes et al., 2021) were analyzed in 17 studies whereas the effects of beta-blockers (Miarons et al., 2016; Miarons et al., 2018) and DPP-4i (Noguchi et al., 2020) were investigated in two and one studies, respectively.

**TABLE 1:** Characteristics of the included studies.

Author, year	Type of pubblication	Study design	Duration (wks)	Drug class	No of patients	Diagnosis	Age			Males (%)
							Range	Mean ± SD	Median (IQR range)	
*Arai et al. (1998a)*	PRA	pOBS	156	ACEi	269	Hypertension	≥65	Males: 75.3 Females: 75.6	NA	46.5
				CCB	247					43.7
				Control	60					NA
*Arai et al. (1998b)*	PRA	CT	12	ACEi	16	Stroke, hypertension and symptomless dysphagia	NA	NA	NA	44
				ACEi	10	Hypertension without dysphagia				60
				Control	7	Healthy				NA
*Arai et al. (2001)*	PRA	pOBS	104	ACEi	209	Stroke and hypertension	NA	NA	NA	47
				ARB	195					50
*Arai et al. (2003)*	PRA	RCT	12	ACEi (various dosages)	13	Stroke and silent aspiration	NA	80 ± 4	NA	48.3
					14			79 ± 5		
					12			79 ± 5		
					9			80 ± 4		
				Control	12			79 ± 5		
*Arai et al. (2005)*	PRA	pOBS	152	ACEi	430	Stroke and hypertension	≥65	75 ± 1	NA	47.9
				CCB	409			75 ± 1		47.9
				Diuretics	351			75 ± 1		47.9
				Control	160			76 ± 1		51.3
*Boschet al. (2012)*	PRA	pOBS	26	ACEi	120	Dementia and aspiration pneumonia	75–95	86 ± 9	NA	49,2
*Fernandes et al. (2021)*	PRA	rOBS	96	ACEi	204	Elderly with different diagnosis	60–89	Males: 68.3 ± 5.9 Females: 70.2 ± 7.2	NA	39.7
*Kano et al. (2016)*	CA	RCT	13	ACEi	19	Amyotrophic lateral sclerosis	NA	NA	NA	NA
Kumazawa et al. (2019)	PRA	rOBS	13	ACEi	5789	Stroke and aspiration pneumonia	≥50	NA	81 (73.5–87)	59.8
				ARB	5789				81 (73–87)	59.9
*Lee et al. (2015)*	PRA	RCT	26	ACEi	33	Cerebrovascular diseases and dysphagia	≥60	83.4 ± 6.8	NA	24.2
				Control	38			84.4 ± 5.6		34.2
*Liu et al. (2012)*	PRA	rOBS	4	ACEi, ARB	13,832	Stroke and pneumonia	≥18	75.2 ± 10.5	NA	63.5
*Marciniak et al. (2009)*	PRA	rOBS	192	ACEi	36	Stroke	≥18	66.1 ± 10.5	NA	61
					36			66.5 ± 13.7		61
Matsumoto et al. (2012)	CA	pOBS	261	ACEi	204	Stroke	NA	NA	NA	NA
*Miarons et al. (2016)*	PRA	rOBS	13	Beta-blockers, CCB, agents acting on the ARB, oral anti-diabetics	966	Elderly with different diagnosis	≥70	Overall: 85.3 ± 6.37	NA	40.7
								Dysphagic: 86.5 ± 6.51		
								Non-dysphagic: 84.6 ± 6.29		
*Miarons et al. (2018)*	PRA	pOBS	36	Beta-blockers	28	Adults with different non-neurological diagnosis	50–80	64.99 ± 1.38	NA	42.9
				Control	28			65.61 ± 1.21	NA	42.9
*Nakashima et al. (2011)*	PRA	RCT	26	ACEi	30	Elderly with different diagnosis, history of pneumonia and dysphagia	≥65	NA	80 (77.0–84.3)	76.7
				Nicergoline	30				79.0 (75.5–83.0)	76.7
*Nakayama et al. (1998)*	PRA	RCT	2	ACEi	22	Normotensive elderly with different diagnosis and aspiration pneumonia	NA	75 ± 2	NA	NA
				Control	10			75 ± 3		
*Noguchi et al. (2020)*	PRA	rOBS	672	DPP4i	NA	Diabetes	NA	NA	NA	NA
*Sato et al. (2013)*	CA	rOBS	83.8 (median)	ACEi, antihypertensive drugs other than ACEi	224	Non-metastatic head and neck cancer	NA	NA	59.6	85.7
*Shimizu et al. (2008)*	PRA	CT	6	ACEi	10	Stroke and dysphagia	NA	70 ± 4	NA	70

PRA, peer-reviewed article; CA, congress abstract; pOBS, prospective Observational Study; CT, Single-arm Clinical trial; RCT; randomized controlled trial; rOBS, retrospective Observational Study; ACEi, Angiotensin-Converting Enzyme Inhibitors; CCB; Calcium-channel blocker; ARB, Angiotensin II, receptor blockers; DPP-4i, Dipeptidyl-Peptidase IV, inhibitors; NA, not available.

#### 3.1.2 Characteristics of the included studies

Studies were published between 1998 and 2021. Most studies (Arai et al., 1998a; Arai et al., 1998b; Nakayama et al., 1998; Arai et al., 2001; Arai et al., 2003; Arai et al., 2005; Shimizu et al., 2008; Nakashima et al., 2011; Liu et al., 2012; Matsumoto et al., 2012; Sato et al., 2013; Lee et al., 2015; Kano et al., 2016; Kumazawa et al., 2019; Noguchi et al., 2020) were from Asian countries; three publications were from European countries (Bosch et al., 2012; Miarons et al., 2016; Miarons et al., 2018); and two publications were from American countries (Marciniak et al., 2009; Fernandes et al., 2021). Seventeen publications were articles in peer-reviewed journals and three were congress abstracts. Concerning study design, seven were retrospective observational studies, six were prospective observational studies, five were RCTs, and two were single-arm CTs. The sample size was <50 in four studies (Arai et al., 1998a; Nakayama et al., 1998; Shimizu et al., 2008; Kano et al., 2016), between 50 and 99 in five studies (Arai et al., 2003; Marciniak et al., 2009; Nakashima et al., 2011; Lee et al., 2015; Miarons et al., 2018) between 100 and 500 in five studies (Arai et al., 2001; Bosch et al., 2012; Matsumoto et al., 2012; Sato et al., 2013; Fernandes et al., 2021) and >500 in five studies (Arai et al., 1998b; Arai et al., 2005; Liu et al., 2012; Miarons et al., 2016; Kumazawa et al., 2019); in one study the overall sample size was not specified (Noguchi et al., 2020). The majority of the studies recruited neurological patients (10 studies on stroke (Arai et al., 1998a; Arai et al., 2001; Arai et al., 2003; Arai et al., 2005; Shimizu et al., 2008; Marciniak et al., 2009; Liu et al., 2012; Matsumoto et al., 2012; Lee et al., 2015; Kumazawa et al., 2019), one study on dementia (Bosch et al., 2012), and one on amyotrophic lateral sclerosis); in five studies participants had multiple etiologies (Nakayama et al., 1998; Nakashima et al., 2011; Miarons et al., 2016; Miarons et al., 2018; Fernandes et al., 2021). One study focused on diabetic (Noguchi et al., 2020) patients, one study included patients with head and neck cancer, and one study recruited patients with hypertension (Arai et al., 1998b). Mean age of recruited patients was >65 years for all the studies, with eight studies including only elderly patients (Arai et al., 1998a; Nakayama et al., 1998; Nakashima et al., 2011; Bosch et al., 2012; Miarons et al., 2016; Fernandes et al., 2021). With regard to the pharmacological intervention, the effects of ACEi (Arai et al., 1998a; Arai et al., 1998b; Nakayama et al., 1998; Arai et al., 2001; Arai et al., 2003; Arai et al., 2005; Shimizu et al., 2008; Marciniak et al., 2009; Nakashima et al., 2011; Bosch et al., 2012; Liu et al., 2012; Matsumoto et al., 2012; Sato et al., 2013; Lee et al., 2015; Kano et al., 2016; Kumazawa et al., 2019; Fernandes et al., 2021) were analyzed in 17 studies, whereas the effects of beta-blockers (Miarons et al., 2016; Miarons et al., 2018) and DPP4i (Noguchi et al., 2020) were investigated in two and one studies, respectively.

#### 3.1.3 Methodological quality of the included studies

Supplementary Figure S3; Supplementary Table S4 show the results of the methodological assessment of included studies using the RoB2 and the ROBINS-I assessment tools. Risk of bias was on average high, with only one study classified as having low risk of bias (Nakashima et al., 2011). Concerning RCTs analyzed with ROB2, risk of bias was classified as low in one study (Nakashima et al., 2011) for two of the three outcomes of interest and high for the remaining outcome, moderate in one study (Lee et al., 2015), and high in three studies (Arai et al., 1998a; Nakayama et al., 1998; Kano et al., 2016). Most critical domains were related to the randomization process and the deviations from intended interventions. At the ROBINS-I, studies were classified as moderate risk of bias in 2 cases (Liu et al., 2012; Kumazawa et al., 2019), serious risk of bias in 2 (Shimizu et al., 2008; Miarons et al., 2016), and critical risk of bias in 5 cases (Marciniak et al., 2009; Bosch et al., 2012; Miarons et al., 2018; Noguchi et al., 2020; Fernandes et al., 2021); in six studies (Arai et al., 1998a; Arai et al., 1998b; Arai et al., 2001; Arai et al., 2005; Matsumoto et al., 2012; Sato et al., 2013) (congress abstracts or letters to the editor) there was not enough information to assess risk of bias. The most critical areas were biases due to deviations from the intended intervention, due to lack of information on, and biases in the classification of interventions.

#### 3.1.4 Effect of pharmacological treatments on dysphagia

The effect of the drugs of interest on swallowing function was assessed in 11 studies (4RCTs, 2 CTs, one prospective observational study, and four retrospective observational studies) (Table 2). In particular, nine studies analyzed the effects of ACEi, two studied the effects of beta-blockers, and one studied the effects of DPP-4i. Dysphagia detection was based on instrumental assessment in five studies (Arai et al., 1998b; Nakayama et al., 1998; Arai et al., 2003; Shimizu et al., 2008; Matsumoto et al., 2012) clinical assessment in four studies (Lee et al., 2015; Miarons et al., 2016; Miarons et al., 2018; Fernandes et al., 2021), and was patient and/clinician-reported in two studies (Noguchi et al., 2020; Fernandes et al., 2021). Only four studies used validated scales for dysphagia (Lee et al., 2015; Miarons et al., 2016; Miarons et al., 2018; Fernandes et al., 2021).

**TABLE 2:** Effects of pharmacological treatments on dysphagia.

Author, year	Study design	Drug of interest	Concomitant therapy	Outcome measures	Assessment methods	% Dysphagia baseline	% Dysphagia post-treatment	Results
ACEi								
Arai et al. (1998b)	CT	Imidapril 5–10 mg/day (patients wtih symptomless dysphagia)	Simultaneous administration of other medications was allowed except for Levodopa	% silent aspiration	instrumental assessment	100%	62.5%	Silent aspiration improved in 10/16 patients after 12 weeks of ACEi
		Imidapril 5–10 mg/day (patients without dysphagia)				0%	0%	
		Control (healthy)				0%	0%	
Nakayama et al. (1998)	RCT	Imidapril 5 mg/day	NA	Duration of swallowing reflex delay	instrumental assessment (sEMG)	100%	NA	The latency of response of the swallowing reflex did not differ between placebo and imidapril in the healthy volunteers (baseline 1.4 ± 0.2 s vs. after treatment 1.2 ± 0.1 s). In patients with aspiration pneumonia, ACEi significantly improved the latency of response compared with placebo (baseline 6.3 ± 1.1 s vs. after treatment 2.7 ± 0.3 s)
		Control (placebo)						
Arai et al. (2003)	RCT	Imidapril 1.25 mg/day	NA	% silent aspiration	instrumental assessment	100%	26.2%	Silent aspiration disappeared in the majority of the patients (31/42) in the treatment group (12 weeks of ACEi), whereas only in 1/12 patient in the control group. Response to treatment (no silent aspiration post-treatment) was proportional to the ACEi dose: 50% for 0.25 mg/day, 73% for 0.5 mg/day, 77% for 0.625 mg/day, 83% for 1.25 mg/day
		Imidapril 0.625 mg/day						
		Imidapril 0.5 mg/day						
		Imidapril 0.25 mg/day						
		Control (no treatment)				100%	91.7%	
Shimizu et al. (2008)	CT	Imidapril 5 mg/day	NA	Pharyngeal transit time	instrumental assessment (VFSS)	100%	NA	The ACE inhibitor decreased the pharyngeal transit time in all 10 patients, resulting in a significant reduction of the pharyngeal transit time after 6-week treatment with the ACE inhibitor (baseline 2.5 ± 0.3 s, after treatment 1.6 ± 0.2 s, *p* < 0.01). The abnormalities in the oral and esophageal stages were not altered by treatment with the ACE inhibitor
Nakashima et al. (2011)	RCT	Imidapril 5 mg/day	Statins, angiotensin receptor blockers, calcium channel blockers, L-dopa and amantadine	% delayed swallowing reflex	clinical assessment	100%	73.3%	Swallowing reflex delay improved in 15/30 (50%) of patients taking imidapril and 19/30 (63.3%) patients taking nicergoline. There was no significant difference in the overall proportion of patients who showed improvements in dysphagia with imidapril compared to nicergoline
		Control (Nicergoline 15 mg/day)				100%	60%	
Matsumoto et al. (2012)	pOBS	Perindopril 2–4 mg/day	Hospital-based conventional rehabilitation program and home-based exercise program	Dysphagia	instrumental assessment (VFSS)	NA	NA	Swallowing function was improved in parallel with the increase of motor outcomes
Lee et al. (2015)	RCT	Lisinopril 2.5 mg/day	NA	RBHOMS score	clinical assessment	100%	NA	At baseline, swallowing function did not differ between the two groups (RBHOMS mean score treatment 3.7 ± 0.8 vs. placebo 4.2 ± 1.5, *p* = 0.462). At week 12, there was a better swallowing function in patients treated with ACEi than placebo (RBHOMS mean score treatment 4.2 ± 1.5 vs. placebo 3.5 ± 1.5, *p* = 0.053)
		Control (placebo)				100%	NA	
Fernandes et al. (2021)	rOBS	ACEi	Anticholinergics, antimuscarinics, antihistamines, antidepressants, antipsychotics, opioids, L-dopa, adrenergics, thyroid hormones, cholinergic drugs, diuretics, sedatives, NSAIDs, corticosteroids, antiulcerogenics, antihypertensive, antidiabetics, antiadrenergics	EAT-10	patient-reported	NA	NA	ACEi users complained more about symptoms of dysphagia. Scores were significantly higher on the EAT-10 than non-users (ACEi users mean EAT-10 = 1.7, non-users mean EAT-10 = 0.7, *p* = 0.038)
ACEi and Beta-blockers								
Miarons et al. (2016)	rOBS	ACEi	Selective calcium channel blockers, anti-inflammatory and antirheumatic products, non-steroids, antipsychotics, antidepressants, drugs against dementia	% dysphagia at the VVST	clinical assessment	NA	NA	ACEis were not associated with potential beneficial actions on swallowing (OR 0.68, 95%CI 0.46–1.02, *p* = 0.060). Beta-blockers were independently associated with a reduced risk of dysphagia (OR 0.60, 95%CI 0.38–0.95, *p* = 0.030)
		Beta-blockers				NA	10.4%	
		Oral antidiabetics				NA	15.1%	
Beta-blockers								
Miarons et al. (2018)	rOBS	Beta-blockers	Drugs for alimentary tract and metabolism, drugs for blood and blood-forming organs, drugs for the cardiovascular system, drugs for genitourinary system and sex hormones, systemic hormonal preparations (excluding sex hormones), anti-infectives for systemic use, drugs for muscoloskeletal system, drugs for nervous system, drugs for respiratory system	% dysphagia at the VVST	clinical assessment	NA	32.1%	Patients taking beta-blockers had a significantly lower frequency of dysphagia than patients not taking beta-blockers
		Control (no beta-blockers)				NA	67.9%	
DPP-4i								
Noguchi et al. (2020)	rOBS	DPP-4i	Sulfonylurea, glinide, biguanide, thiazolidinedione, *a*-glucosidase inhibitors, glucagon-like peptide-1 receptor agonist, sodium glucose cotransporter-2 inhibition	dysphagia as adverse event	patient or physician reported	NA	NA	7 cases of DPP-4i induced dysphagia were found. No significant association between DPP4i use and dysphagia was detected (ROR: 0.82, 95%CI: 0.39–1.73)

CT, Single-arm Clinical trial; RCT; randomized controlled trial; pOBS, prospective Observational Study; rOBS, retrospective Observational Study; ACEi, Angiotensin-Converting Enzyme Inhibitors; DPP-4i, Dipeptidyl-Peptidase IV, inhibitors; RBHOMS, royal brisbane hospital outcome measure for swallowing; EAT-10, Eating Assessment Tool; VVST, Volume-viscosity swallow test; sEMG, surface electromyography; VFSS, videofluoroscopic swallowing study; NA, not available.

ACEi was the main drug class studied. Six studies reported an improvement of dysphagia in the majority of the patients treated with ACEi (Arai et al., 1998a; Nakayama et al., 1998; Arai et al., 2003; Shimizu et al., 2008; Nakashima et al., 2011; Matsumoto et al., 2012; Lee et al., 2015). Additionally, Arai and others (Arai et al., 2003) compared the efficacy of ACEi at different doses and detected a dose-response relationship with the improvement of dysphagia. Improvements in swallowing function targeted the pharyngeal phase of swallowing and included a reduction of the rate of silent aspiration (Arai et al., 1998b; Arai et al., 2003), pharyngeal transit time (Shimizu et al., 2008), and swallowing reflex delay (Nakayama et al., 1998; Nakashima et al., 2011). On the contrary, one study on elderly patients with different etiology failed to detect any association between ACEi use and dysphagia (Miarons et al., 2016), and another study, on a similar population, found that ACEi users complained of more dysphagia symptoms than non-users (Fernandes et al., 2021). However, the mean score on the self-reported Eating Assessment Tool (EAT-10) (Belafsky et al., 2008), used to assess dysphagia in the latter study, was lower than the cut-off for dysphagia (EAT-10 ≥ 3) in both groups.

Two retrospective observational studies by Miarons and others (Miarons et al., 2016; Miarons et al., 2018) reported an independent association between beta-blocker use and reduced risk of dysphagia. This association was clinically detected using a validated clinical assessment protocol (Clavé et al., 2008) in elderly patients with different neurological and non-neurological diseases. However, no data on the frequency of dysphagia before the pharmacological treatment is available because of the retrospective nature of the studies.

Only one study investigated the effects of DPP-4i on swallowing function (Noguchi et al., 2020). Based on the adverse events from a spontaneous report system, Noguchi and others found no association between dysphagia and the use of DPP-4i in diabetic patients.

#### 3.1.5 Effect of pharmacological treatments on aspiration pneumonia

The effect of the drugs of interest on aspiration pneumonia was assessed in 12 studies (2RCTs, four prospective observational studies, and six retrospective observational studies) (Table 3). In particular, 11 studies analyzed the effects of ACEi and one study analyzed the effects of DPP-4i. Four studies focused only on aspiration pneumonia (Marciniak et al., 2009; Bosch et al., 2012; Lee et al., 2015; Kumazawa et al., 2019), while the remaining studies focused on pneumonia in general, including aspiration pneumonia, in patients with documented dysphagia or who were at a high risk of dysphagia.

**TABLE 3:** Effects of pharmacological treatments on first or recurrent aspiration pneumonia rate.

Author, year	Study design	Drug of interest	Concomitant therapy	Definition of aspiration pneumonia	% 1st aspiration pneumonia	% Recurrent aspiration pneumonia	Results
ACEi							
Arai et al. (1998a)	pOBS	Imidapril hydrochloride	NA	NA	3.3%	NA	The rate of pneumonia in the ACEi group was significantly lower than in the calcium-channel blocker group (*p* = 0.025)
		Calcium-channel blocker			8.9%	NA	
		Control (non-hypertensive patients)			8.3%	NA	
Arai et al. (2001)	pOBS	ACEi	NA	NA	4.4%	NA	The incidence of pneumonia in the ACEi group was significantly lower than the ARB group (*p* = 0.013)
		ARB			11.2%	NA	
Arai et al. (2005)	pOBS	ACEi	NA	NA	2.8%	NA	Patients treated with ACEi had a lower risk of pneumonia than controls (hazard ratio 0.30, 95% CI 0.14–0.66, *p* = 0.001)
		Calcium-channel blocker			8.8%	NA	
		Diuretics			8.3%	NA	
		Control (no antihypertensive drugs)			8.8%	NA	
Marciniak et al. (2009)	rOBS	ACEi	PPI, H2 blockers	Clinical setting of fever, chills, muscle stiffness, chest pain, cough, shortness of breath, rapid heart rate, or difficulty breathing, with chest x-ray confirmation either in rehabilitation or after transfer to acute care	NA	NA	Use of ACE inhibitors was similar for both stroke patients with pneumonia and matched-controls; ACE inhibitors did not confer any decreased risk of pneumonia (OR, 0.9; 95% CI, 0.2–3.0)
Nakashima et al. (2011)	RCT	Imidapril 5 mg/day	Statins, angiotensin receptor blocker, calcium channel blocker, L-dopa and amantadine	Pneumonia was diagnosed based on the Japanese Respiratory Society guidelines	Inclusion criteria	30%	No significant difference in the pneumonia recurrence rate was found between the imidapril and the nicergoline groups
		Nicergoline 15 mg/day			Inclusion criteria	16.7%	
Bosch et al. (2012)	pOBS	ACEi	Psychotropic drugs, histamine receptor blocker or PPI treatment in the mont previous to admission, neuroleptics, SSRI, antibiotics before hospitalization	Infiltrate on chest radiography consistent with pneumonia and one major	NA	NA	Patients with recurrent aspiration pneumonia were less-frequently prescribed ACEi compared with a first episode of aspiration pneumonia (8.8% vs. 27.9%, *p* < 0.001)
				symptom or sign (cough, sputum production or temperature above 37.8°C) or two minor criteria (dyspnea, pleuritic chest pain, delirium, respiratory rate>20 bpm, signs of pulmonary consolidation, or leukocyte count>12 × 109/L). In addition, to meet our definition of AP, all patients had to have risk factors for oropharyngeal aspiration and a history of witnessed or suspected aspiration			
Liu et al. (2012)	rOBS	ACEi	Statins, PPI, histamine type 2 receptor antagonists	ICD9-CM: 507 pneumonitis due to solids and liquids, 481 Pneumococcal pneumonia	Inclusion criteria	NA	ACEi use was associated with a lower pneumonia risk (OR 0.77; 95%CI 0.68–0.87). An increased mean defined daily dose (DDD) was associated with significantly reduced pneumonia risk (DDD>1 mg daily)
				482 Other bacterial pneumonia			
				483 Pneumonia due to other specified organism, 485 Bronchopneumonia, organism unspecified			
				486 Pneumonia, organism unspecified			
Matsumoto et al. (2012)	pOBS	Perindopril 2–4 mg/day	Hospital-based conventional rehabilitation program and home-based exercise program	NA	2.9%	NA	6 events of pneumonia were recorded over a 5-year period
Sato et al. (2013)	rOBS	ACE-i	Chemoradiotherapy (cisplatin, carboplatin, docetaxel)	NA	0%		Patients with ACEi had a lower rate of aspiration pneumonia compared to patients with other antihypertensive drugs or patients without antihypertensive drugs (0% vs. 17.8% vs. 12.5%; no statistical analysis was performed)
		Antihypertensive drugs other than ACE-i			17.8%		
		No antihypertensive drugs			12.5%		
Lee et al. (2015)	RCT	Lisinopril 2.5 mg/day	NA	Presence of new pneumonic changes in the chest x-ray (done in index admission used for References) and 1 major clinical sign: increased	57.6%	NA	The incidences of pneumonia and fatal pneumonia (pneumonia-related death) were not significantly different between the groups (pneumonia: treatment 57.6% vs. placebo 47.4%, *p* = 0.390; fatal pneumonia: treatment 42.4% vs. placebo 26.3%, *p* = 0.152)
		Control (placebo)		sputum production or 2 of the following minor clinical signs: raised or depressed white cell count, hypoxia at room air (SpO2 <92%) and tympanic temperature greater than 38 °C	47.4%	NA	
Kumazawa et al. (2019)	rOBS	ACEi	Antipsychotic, anti-dementia drugs, antiemetics, antiepileptics, antitussive drugs, muscle relaxants, antidiabetics, steroids, immunosuppressive drugs, gastric secretion inhibitors, antidyslipidemics, antithrombotics, antihypertensive, diuretics, amantadine, nicergoline, severe pneumonia antibiotics	ICD-10 codes: Aspiration Pneumonia (J69) and bacterial pneumonia (J13-J18)	Inclusion criteria	0.8% at 14 days 1.3% at 30 days 2.6% at 90 days	Non-significant difference was seen in 14-day, 30-day, or 90-day post-stroke readmission for aspiration pneumonia between patients on ACEi and patients on ARB.
		ARB			Inclusion criteria	0.7% at 14 days 1.3% at 30 days 2.4% at 90 days	
DPP-4i							
Noguchi et al. (2020)	rOBS	DPP-4i	Sulfonylurea, glinide, biguanide, thiazolidinedione, *a*-glucosidase inhibitors, glucagon-like peptide-1 receptor agonist, sodium glucose cotransporter-2 inhibition	NA	NA	NA	35 cases of DPP-4i induced aspiration pneumonia were found. DPP-4i use was significantly associated with an increased risk of aspiration pneumonia (ROR 1.67, 95%CI: 1.20–2.34). When DPP-4is were analyzed individually, a significant association with aspiration pneumonia risk was detected for trelagliptin (ROR 9.99, 95%CI: 4.10–24.36), linagliptin (ROR 2.66, 95% CI: 1.19–5.94) and sitagliptin (ROR 1.84, 95% CI: 1.04–3.25)

pOBS, prospective Observational Study; rOBS, retrospective Observational Study; RCT; randomized controlled trial; ACEi, Angiotensin-Converting Enzyme Inhibitors; ARB, Angiotensin II, receptor blockers; DPP-4i, Dipeptidyl-Peptidase IV, inhibitors; NA, not available; PPI; proton pump inhibitor; SSRI, selective serotonin reuptake inhibitors; ICD, international classification of diseases; DDD, defined daily dose; OR, odds ratio; ROR; relative odds ratio; CI, confidence interval.

Six studies reported results indicating the beneficial effects of ACEi on aspiration pneumonia risk (Arai et al., 1998a; Arai et al., 2001; Arai et al., 2005; Bosch et al., 2012; Liu et al., 2012; Sato et al., 2013). ACEi use was associated with a lower rate of pneumonia compared to no treatment (Arai et al., 2005; Sato et al., 2013) or to other hypertensive drugs (Angiotensin II receptor blockers, Calcium-channel blockers). One study suggested a dose-response relationship in reducing pneumonia risk (Liu et al., 2012). Additionally, one study found a protective effect of ACEi on the recurrence of aspiration pneumonia (Bosch et al., 2012). Conversely, four studies failed to detect an association between ACEi use and reduced aspiration pneumonia risk (Nakashima et al., 2011; Liu et al., 2012; Lee et al., 2015; Kumazawa et al., 2019). In these studies, the incidence of the first pneumonia event or pneumonia recurrence did not differ between patients treated with ACEi and patients treated with Angiotensin II receptor blockers (Kumazawa et al., 2019), nicergoline (Nakashima et al., 2011), other drugs or placebo (Lee et al., 2015).

The only study investigating the effects of DPP-4i on aspiration pneumonia based on a spontaneous report system of adverse events found an increased risk of aspiration pneumonia in diabetic patients treated with DPP-4i (Noguchi et al., 2020).

#### 3.1.6 Effect of pharmacological treatments on substance P concentration

The effects of the drugs of interest on SP concentration was assessed in five of the included studies (3RCTs, and one retrospective observational study) (Table 4). Among them, four studies analyzed the efficacy of ACEi, and one study focused on beta-blockers. All the studies on ACEi reported a significant increase in mean serum SP levels in patients treated with ACEi (Arai et al., 1998b; Arai et al., 2003; Nakashima et al., 2011; Kano et al., 2016). Improvements in serum SP concentrations have been associated with improvements in swallowing safety (Arai et al., 1998a; Arai et al., 2003; Nakashima et al., 2011). However, this correlation may not always be straightforward. A minority of the patients who did not improve their swallowing function were found to have increased SP concentrations and, on the contrary, some of the patients who did improve their swallowing function did not record an increase in SP (Arai et al., 1998b; Arai et al., 2003).

**TABLE 4:** Effects of pharmacological treatments on serum substance P concentration.

Author, year	Study design	Drug of interest	Concomitant therapy	Serum SP baseline (pg/mL)	Serum SP post-treatment (pg/mL)	Results
				Mean ± SD	Mean ± SD	
Studies on ACEi						
Arai et al. (1998b)	CT	Imidapril 5–10 mg/day (patients with symptomless dysphagia)	Simultaneous administration of other meds was allowed except for L-dopa	23.3	NA	Serum SP concentrations at the baseline were lower in patients with dysphagia than the other groups. After treatment, serum SP concentrations increased in 8/10 patients who improved swallowing function (post-treatment mean 79.3 pg/mL, baseline mean 23.3 pg/mL), whereas did not change in the remaining 2/10 responders. Among patients who did not improve swallowing function, 3/5 showed an increase in SP concentration (mean 82.1 pg/mL), while 2/5 had no change
		Imidapril 5–10 mg/day (patients without dysphagia)		76.5	NA	Serum SP did not change from baseline
		Control (healthy)		72.7	NA	NA
Arai et al. (2003)	RCT	Imidapril	NA	26.0 ± 1.7 (patients with swallowing improvement) 26.4 ± 1.1 (patients with no swallowing improvement)	68.8 ± 6.0 (patients with swallowing improvement) 45.4 ± 8.6 (patients with no swallowing improvement)	Serum SP levels significantly increased patients treated with ACEi, regardless of the improvement in silent aspiration, whereas did not increase in the control group. Serum SP levels at the end of the study were significantly higher: (i) in patients treated with ACEi who improved swallowing function than in patients treated with ACEi who did not improve swallowing function; (ii) in patients treated with ACEi than controls
		1.25 mg/day				
		Imidapril				
		0.625 mg/day				
		Imidapril				
		0.5 mg/day				
		Imidapril				
		0.25 mg/day				
		Control (no treatment)		26.4 ± 1.0	25.3 ± 1.1	
Nakashima et al. (2011)	RCT	Imidapril	Statins, angiotensin receptor blocker, calcium channel blocker, L-dopa and amantadine	NA	NA	Both imidapril and nicergoline significantly increased serum levels of SP, with no significant differences among the two groups. Patients whose dysphagia was improved showed significantly increased serum levels of SP. By contrast, the patients whose dysphagia failed to improve did not show significant increases in serum levels of SP.
		5 mg/day				
		Nicergoline		NA	NA	
		15 mg/day				
Kano et al. (2016)	RCT	Enalapril	NA	NA	NA	At 3 months, sputum SP concentration increased in ALS patients treated with enalapril
		5 mg/day				
		Control (no treatment)		NA	NA	
Studies on beta-blockers						
Miarons et al. (2018)	rOBS	Beta-blockers	Drugs for alimentary tract and metabolism, blood and blood-forming organs, cardiovascular system, genitourinary system and sex hormones, systemic hormonal preparations, musculoskeletal system, nervous system, respiratory system	NA	260.68 ± 144.27	SP serum levels were significantly higher in patients taking beta-blockers than in patients not taking beta-blockers
		Control (no beta-blockers)		NA	175.46 ± 108.36	

SD, standard deviation; SP, substance P; ACEi, Angiotensin-Converting Enzyme Inhibitors; CT, clinical trial; RCT, randomized controlled trial; NA, not available; rOBS, retrospective observational study.

Miarons and others detected significantly higher serum and saliva SP levels in adult non-neurological patients taking beta-blockers compared to patients not taking beta-blockers, matched for age, sex, and independence level (Miarons et al., 2018). However, due to the cross-sectional design of the study, no association between treatment with beta-blockers and improvement in SP concentrations can be determined.

#### 3.1.7 Adverse events

Four studies reported adverse events related to the treatment with ACEi. Three studies reported excessive cough in 5.3%–6.3% of the patients (Arai et al., 1998a; Arai et al., 2003; Kano et al., 2016), in one study pneumonia onset was associated with the treatment with ACEi in 6.3% of the patients (Arai et al., 2003), and one study reported dizziness or hypotension in 5.3% of the patients (Kano et al., 2016). Regarding serious adverse events, one RCT was prematurely terminated due to significantly higher mortality in the intervention group at interim analysis (Lee et al., 2015).

### 3.2 Pharmacovigilance study

From the FAERS we identified 1,742,491 ICSRs involved elderly subjects. 1,453,966 ICSRs came from North America and Europe; of these, 12,302 ICSRs (0.8%) were related to dysphagia/aspiration pneumonia. The descriptive analysis of demographic data and the characteristics of nested-cases and non-cases populations is presented in Table 5. The median age was significantly different between cases and non-cases (*p* < 0.05); however, most of the patients were 70–80 years old (25th–75th percentiles, 70–82 vs. 69–80 for cases and non-cases, respectively). In both groups, the percentage of female reports were >50%, *p* < 0.05. Concomitant medications associated with dysphagia were used in 35% of cases and in 22% of non-cases (*p* < 0.05). Dysphagia was mostly reported by consumers (42%). ICSRs were mainly from North America in both cases and non-cases (71%–75%).

**TABLE 5:** Descriptive analysis of the Individual Case Safet Reports (ICSRs) retrieved from FAERS.

	Cases (Dysphagia/Aspiration pneumonia)	Non-cases (other AEs)
	(*n* = 12,302)	(*n* = 1,441,664)
Age, yrs		
**Mean (SD)**	76 (8)	75 (7)
**Median (25**th**-75**th **percentiles)** **^a^**	75 (70–82)	74 (69–80)
**Age classes n (%)** **^b^**		
**65–75**	6,415 (52)	836,537 (58)
**76–85**	4,217 (34)	465,872 (32)
**86–95**	1,560 (13)	132,800 (9)
**>95**	110 (0.9)	6,455 (0.4)
**Gender, n (%)** **^b^**		
**Females**	6,300 (51)	798,178 (55)
**Concomitant drugs known for risk of Dysphagia/Aspiration Pneumonia, n (%)** **^b^**		
**Yes**	4,295 (35)	316,038 (22)
**Reporter, n(%)** **^b^**		
**Consumers**	5,152 (42)	612,272 (43)
**Healthcare Professionals**	488 (4)	52,955 (4)
**Medical Doctors**	2,789 (23)	329,202 (23)
**Others**	2,054 (17)	237,128 (17)
**Pharmacists**	1,252 (10)	155,251 (11)
**Not Available**	567 (5)	54,856 (4)
**Country** **^b^**		
**North America**	8,677 (71)	1,088,217 (75)
**Europe (Western/Southern/Northern Countries)**	3,625 (29)	353,447 (25)

^a^ *p* < .05, Mann-Whitney *U* test.

^b^ *p* < .05, chi-squared test.

AEs: Adverse Events.

#### 3.2.1 Disproportionality analysis

Results are presented in Table 6. Ranked by the absolute number of reports, the highest number of outcomes of interest were reported for Levodopa (*n* = 810), followed by Levodopa associated with beta-blockers (*n* = 82) and ACEi (*n* = 51). Sacubitril could not be included in the analysis because, even if five non-cases were present, no ICSRs reporting dysphagia were retrieved. Since all the other drugs were chosen as the Reference group, results must be read in comparison with them.

**TABLE 6:** Univariate and multivariate logistic regression.

	Dysphagia/Aspiration pneumonia (*n* = 12,302)	Other AEs (*n* = 1,441,664)	cROR (95% CI)	aROR (95% CI)^a^
***Over 65 patients***	*11,317*	*1,418,163*	*Ref. Group*	*Ref. Group*
***Levodopa***	810	18,366	5.5 (5.1; 5.9)^b^	4.8 (4.5; 5.2)^b^
***Levodopa + ACE inhibitors***	51	1,295	4.9 (3.7; 6.5)^b^	3.6 (2.7; 4.7)^b^
***Levodopa + β-blockers***	82	2,696	3.8 (3.0; 4.7)^b^	2.8 (2.2; 3.4)^b^
***Levodopa + Gliptins***	3	165	2.3 (0.6; 6.0)	1.8 (0.4; 4.7)
***Levodopa + More than one drug of interest***	39	979	5.0 (3.6; 6.8)^b^	3.6 (2.6; 4.9)^b^

^a^ Adjustment for age class, gender and other concomitant drugs known to increase the risk of dysphagia.

^b^ *p* < 0.001.

Italic values refer to ICSRs where none of the drugs of interest were reported; these ICSRs were used as reference group, the remaining results must be read in comparison with them.AEs: Adverse Events; aROR: adjusted Reporting Odds Ratio; cROR: crude Reporting Odds Ratio.

As expected, compared to the general population over 65, which is known to be more at risk for dysphagia and aspiration pneumonia, patients treated with only Levodopa had the highest and most significant ROR, even after adjustment [aROR (95%CI) = 4.8 (4.5; 5.2)]. Then, in general, the concomitant use of drugs inhibiting the degradation of SP reduces the reporting risk of Levodopa: ACEi [aROR (95%CI) = 3.6 (2.7; 4.7)] and studies with more than one drug of interest [aROR (95%CI) = 3.6 (2.6; 4.9)], followed by beta-blockers [aROR (95%CI) = 2.8 (2.2; 3.4)], and gliptin s reaching a non-significant difference from the Reference group [aROR (95%CI) = 1.8 (0.4; 4.7)].

The planned sensitivity analysis involving only ICSRs reported by physicians (total sub-cohort: 331, 991 ICSRs) was not performed because, among 2,789 ICSRs reporting dysphagia, no cases reported Levodopa associated with DPP-4i; 26 ICSRs reported Beta-blockers (9% vs. 6% of cases in the main analysis) and 19 ACEi (6% vs. 4% of cases in the main analysis).

## 4 Discussion

Drug repositioning has many advantages that make it an attractive drug discovery strategy. First, it simplifies regulatory procedures because clinical data concerning the safety and toxicity of the drug have already been acquired; the development is, therefore, faster and cheaper than *de novo,* and the drug is more likely to be introduced on the market (Ashburn and Thor, 2004).

Our study depicts the current knowledge on the effects of three drug classes (ACEi, beta-blockers, and DPP-4i) on dysphagia and aspiration pneumonia through a systematic review of 20 studies and a real-word data analysis from the spontaneous reporting system database FAERS. Here we will discuss main findings separately, for each drug class included in our focus.

### 4.1 ACE inhibitors

In our systematic review, seven studies reported improvements in dysphagia (Arai et al., 1998b; Nakayama et al., 1998; Arai et al., 2003; Shimizu et al., 2008; Marciniak et al., 2009; Nakashima et al., 2011; Matsumoto et al., 2012; Lee et al., 2015), whereas two studies found no changes or even a worsening in swallowing outcome (Brown, 2013; Fernandes et al., 2021). Differences may be related to the study population and design. All except two of the studies that showed favourable results were conducted on stroke patients. Dysphagia in patients with stroke is often characterized by delayed swallowing reflex, impaired protection of the lower airways, and absent cough reflex (Warnecke et al., 2021). Therefore, the improvement of these conditions seems to be the mechanism behind the protective effect of ACEi (Van de Garde et al., 2007).

The studies that failed to show any positive effects of ACEi on dysphagia were on elderly subjects. The pathophysiological mechanisms underlying dysphagia may be different and, consequently, may not be targeted by ACEi. Additionally, the studies reporting an improvement of dysphagia were all Asian studies. It has been suggested that the effect of ACEi in preventing aspiration pneumonia may be different in Asian and non-Asian populations (Ohkubo et al., 2004; Liu et al., 2012). It was hypothesized that the differential distribution of the ACE insertion/deletion polymorphisms between Asian and non-Asian populations may influence the efficacy of ACEi in improving cough reflex. However, the reasons behind the different effects are still not clearly understood and may be related to differences in the study design or to confounding variables. Finally, two of the seven studies with positive ACEi effects were not controlled (Arai et al., 1998a; Shimizu et al., 2008), thus, the causal relationship between the pharmacological treatment and the positive evolution of dysphagia could not be determined, as spontaneous recovery of swallowing function can occur in stroke patients.

Concerning the effect of ACEi on aspiration pneumonia, the literature is divided between studies showing a protective effect of ACEi (Arai et al., 1998a; Arai et al., 2001; Arai et al., 2005; Bosch et al., 2012; Liu et al., 2012; Sato et al., 2013) and studies failing to detect one when compared to controls or to other pharmacological treatments (Marciniak et al., 2009; Nakashima et al., 2011; Lee et al., 2015; Kumazawa et al., 2019). The only two RCTs that reported no reduction of aspiration pneumonia rate in patients treated with ACEi, but the studies were underpowered due to a small sample size (Nakashima et al., 2011; Lee et al., 2015). The high heterogeneity of the populations and the observational nature of the remaining studies limit the possibilities of comparing and interpreting the results.

Concerning the underlying mechanisms of action, two main mechanisms have been hypothesized: the inhibition of angiotensin II immunomodulatory effect, which reduces pro-inflammatory cytokine release, and the inhibition of the metabolism of both SP and bradykinin, which enhance the swallowing and cough reflexes (Raiden et al., 2002; Arai et al., 2003; He et al., 2007). As the prevention of the degradation of SP induced by ACEi causes its accumulation in the upper respiratory tract and induces the cough reflex (Noguchi et al., 2020), these drugs may then improve swallowing reflexes in patients with a history of aspiration pneumonia (Sekizawa et al., 1998; Okaishi et al., 1999. Although both ACEi and Angiotensin II Receptor Blockers (ARBs) inhibit angiotensin II activity, only ACEi has been shown to shorten the pharyngeal transit time through the increase of substance P and bradykinin levels, improving symptomless dysphagia (Holas et al., 1994; Arai et al., 2000; Shimizu et al., 2008). This activity may suggest that the enhanced cough reflex is most likely the mechanism responsible for the protective effect of ACEi on pneumonia (Liu et al., 2012; Kumazawa et al., 2019). However, there may be other mechanisms (e.g., immune-modulating effect) impacting aspiration pneumonia risk (Suzuki et al., 2003; He et al., 2006; Arndt et al., 2006).

There were some AEs associated with the use of ACEi. The most frequently reported AE was excessive dry cough and was associated with local increase in SP by the inhibition of ACE (Sekizawa et al., 1996). Although the frequency of excessive dry cough was limited to a small percentage of patients (5%–6%) in the retrieved studies (Arai et al., 1998b; Arai et al., 2003; Kano et al., 2016), its occurrence should be monitored as it may interfere with adherence to treatment. One RCTs by Lee et al. was prematurely interrupted because of the high mortality rate in the ACEi group (Lee et al., 2015). Patients in this study were particularly frail, being elderly, tube-fed, and with severe dysphagia, all well-known risk factors for aspiration pneumonia (Palmer and Padilla, 2022). Therefore, the use of ACEi to prevent aspiration pneumonia does not seem to be beneficial in frail high-risk patients and a cautious use of these drugs is recommended to avoid systemic effects on blood pressure, and cardiovascular and renal systems (Cheng et al., 2022).

In the FAERS, ACEi resulted in the highest aROR after Levodopaalone [aROR (95%CI) = 3.6 (2.7; 4.7)]. The data reflect the discordant effect shown by the studies we retrieved through the systematic review: a clear reduction in the ROR was present but less effective than the other drug classes.

### 4.2 Beta-blockers

Findings supporting a potential positive effect of beta-blockers on dysphagia are relatively novel. Only two observational studies from Miarons and others (Miarons et al., 2016; Miarons et al., 2018) were retrieved through our systematic literature review. The authors reported an independent protective effect of beta-blockers on dysphagia in a cohort of elderly subjects with different neurological and non-neurological diseases. Nevertheless, the high refusal rate to participate in the study represents a significant limitation. Future RCTs with adequate sample size are necessary to confirm the efficacy of beta-blockers on dysphagia.

The mechanism by which beta-blockers could exert a protective effect on dysphagia is unknown. Previous research has hypothesized that SP could play a role, as seen with propranolol in guinea pigs (Belvisi, 1996; Lin and Lai, 1998) and supported by Miarons and colleagues’ research in the elderly (Miarons et al., 2018). It seems that beta-blockers prevent the occurrence of dysphagia through the release of pharyngeal SP. Another potential mechanism that has been hypothesized is an increase in contractile forces in pharyngeal muscles because of the upregulation of fast skeletal muscle beta-adrenergic receptors mediated by chronic beta2-adrenergic blockade (Murphy et al., 1997; Miarons et al., 2016).

Beta-blockers were the most reported drugs in our FAERS analysis, but they were ranked after the ACEi, compared to the reference group, thus suggesting a lower risk of dysphagia for beta-blockers compared to ACEi. Indeed, a recent study on the prevalence of oropharyngeal dysphagia in geriatric patients found a slightly higher prevalence of dysphagia in patients being treated with beta-blockers than among users of ACEi (Wolf et al., 2021). Unfortunately, the analyses of diseases and drugs were separated. It is therefore not possible to directly compare these results with our FAERS analysis since it only covers patients treated with Levodopa and drugs inhibiting the degradation of substance P (Wolf et al., 2021).

### 4.3 DPP-4 inhibitors

SP and GLP-1, a glucose-dependent insulinotropic peptide, are substrates of DPP-4, which is conversely inhibited by the class of antidiabetic drugs known as DPP-4i or gliptins (Noguchi et al., 2020). Since DPP-4i seem to prevent the degradation of SP (Cunningham and O'Connor, 1997; Brown et al., 2009), they were hypothesized to improve swallowing reflex and prevent dysphagia and aspiration pneumonia (Noguchi et al., 2020). Indeed, there have been studies on the potential of diabetic drug repurposing in patients with Parkinson’s disease (Labandeira et al., 2022). A case-control study showed a significant decrease in the incidence of Parkinson’s disease in diabetic patients treated with DPP-4i (OR = 0.23; 95% CI: 0.07–0.74) (Svenningsson et al., 2016) and a similar result was found in a longitudinal cohort study (incidence rate ratio 0.64; 95% CI: 0.43–0.88; *p* < 0.01) (Brauer et al., 2020). Jeong et al. observed a beneficial effect of DPP4-i in a small group of diabetic patients with Parkinson’s disease: they found a higher baseline dopamine transporter availability and better motor performance compared to non-diabetic patients (Jeong et al., 2021). However, even in this specific population, literature concerning the effect of DPP-4i on dysphagia and aspiration pneumonia is very limited. In our systematic review, only one study was retrieved, and it was based on the Japan Adverse Drug Event Report, a Japanese spontaneous pharmacovigilance database (Noguchi et al., 2020). The authors analyzed the events of dysphagia and aspiration pneumonia reported from eight anti-diabetic drugs classes. They concluded that there was no effect of DPP-4i on dysphagia, whereas their use was associated with an increased risk of aspiration pneumonia (Noguchi et al., 2020). Indeed, DPP-4 is the same substance as cell membrane surface antigen CD26, which is also expressed in Tcells (Alexandraki et al., 2006; Reinhold et al., 2007). For this reason DPP-4i may affect the immune system, increasing the risk of developing infections (Willemen et al., 2011).

The data from our analysis on the FAERS contrasts with the results by Noguchi and others (Noguchi et al., 2020).

Differences in the findings may be ascribed to differences in the populations targeted by the pharmacovigilance database search. Indeed, Noguchi et al. focused on diabetic patients, whereas in our analysis we included neurological patients with Parkinsonism being treated with L-dopa. Whereas in Parkinson’s disease a reduction of SP has been associated with the presence of dysphagia (Schröder et al., 2019), in diabetes it might be considered a secondary effect of autonomic neuropathy, which mainly leads to a hyperactivity of the cricopharyngeal muscle and a consequent relaxation of the upper oesophageal sphincter during swallowing (Restivo et al., 2006). Finally, the number of cases reported showing dysphagia and aspiration pneumonia associated with the use of DPP-4i was small both in our analysis and in the study by Noguchi. Therefore, there is a need for prospective studies to clarify the effects of DPP-4i on dysphagia and aspiration pneumonia.

### 4.4 Strengths and limits

This is the first study aimed at exploring the effects of some routinely used drugs on dysphagia and aspiration pneumonia through a combined approach, *i.e*., systematic review and real-world data analysis, to provide the most comprehensive overview of current knowledge on the topic.

The FAERS is the largest repository of spontaneously reported AEs; therefore, it allows access to very large samples, enabling to researchers to elucidate associations between drugs and reported adverse events that would be difficult to investigate with clinical trials.

However, the use of a pharmacovigilance database has some intrinsic limitations. Reporting might be influenced by factors such as notoriety bias, selection bias, and under-reporting, which precludes making causal inferences except in unusual circumstances (Faillie, 2019). As the FAERS is designed to report AEs, unintended positive effects of the drugs on swallowing function could not be recorded.

Furthermore, since case and non-case studies are drawn from different populations, this method cannot be a real substitute for the classical case-control study (Carnovale et al., 2019b; Faillie, 2019; Garcia et al., 2021). The actual risk and incidence rates cannot be determined from the analysis of AE reporting since the primary goal of a spontaneous reporting system is to signal the existence of a possible relationship between therapies and adverse events, without proving any causality.

With regard to the systematic review, since we included both peer-reviewed articles and congress abstracts, the methodological quality of the studies was generally low and the access to the information was limited. Additionally, the population and the outcome measures investigated in the included studies were heterogeneous.

Finally, the mechanisms responsible for the effects of the investigated drugs on swallowing function are still unclear. As described above, a hypothesized common mechanism is the increase of SP levels, being a secondary target for all the drug classes of interest in the present study. The most well-known function of this neuropeptide is the modulation of pain perception (Zieglgänsberger, 2019), but it is also involved in inflammation (Maggi, 1997) and gastrointestinal functions (Saito et al., 2003). With regard to swallowing function, SP stimulates the production of saliva and amylase through a vasodilatory effect in salivary glands (Pikula et al., 1992). In the oropharynx, SP is released by the sensory terminals of the receptors in the pharyngeal mucosa in response to mechanical, thermal and chemical stimuli (Alvarez-Berdungo er al, 2016). As a result, it enhances swallowing and cough reflexes (Jin et al., 1994; Imoto et al., 2011; Canning et al., 2014). Low concentration of SP has been reported in patients with dysphagia (Schröder et al., 2019) and aspiration pneumonia (Nakagawa et al., 1995) and has been associated with reduced spontaneous swallowing frequency (Niimi et al., 2018) and pharyngeal sensitivity (Tomsen et al., 2022). However, the retrospective nature of the case and non-case studies and the lack of data on SP concentrations in the majority of the studies in the systematic review limits the possibility of drawing conclusions on the causal relationship between the increase of SP levels and the protective effects on dysphagia reported in studies on ACEi and beta-blockers. Indeed, other mechanisms, including the primary mechanism of action of these drugs, may be responsible for the positive effects on swallowing function. This might also be in line with the heterogeneity of results in the systematic review when comparing different diseases: instead of a common mechanism of action, the same drug could exert its role in alternative ways in every pathophysiology. Thus, high-quality randomized controlled studies are required both to verify the efficacy of the investigated drugs on dysphagia and its pulmonary complications, and to analyze the role of SP.

## 5 Conclusion

Our study explores the potential repurposing of ACEi**,** beta-blockers, and DPP-4i in neurological patients with dysphagia to improve swallowing function and reduce aspiration pneumonia risk. Although a weak role for SP was hypothesized as one of the potential mechanisms associated with the protective effect on dysphagia, currently available data is insufficient to support this hypothesis.

Due to the nature of the study, no firm conclusion can be drawn on the role of these drugs in effectively ameliorating dysphagia or aspiration pneumonia. Their efficacy and the mechanisms of action should be verified in future high-quality randomized controlled studies. Nevertheless, caution is always required in frail patients at a high risk of pneumonia because of other systemic effects. Further high-quality RCTs, especially from non-Asian countries, are needed to verify the protective effects and identify best responders.

## Data Availability

The raw data supporting the conclusions of this article will be made available by the authors, without undue reservation.
