# Supplementary material for: On the potential of drug repurposing in dysphagia treatment: New insights from a real-world pharmacovigilance study and a systematic review
Source: Front Pharmacol. 2023 Mar 3;14:1057301. doi: 10.3389/fphar.2023.1057301 (PMC10022593; doi:10.3389/fphar.2023.1057301)
Supplement: Supplementary file 1 [file Table1.DOCX]

Supplementary Material

**S1. Electronic Databases Search Strategy**

**Pubmed**

(("Hypoglycemic Agents"[Mesh] OR “antidiabetic” [tiab] OR “Hypoglycemic Agent” [tiab] OR “Antihyperglycemic Agent” [tiab] OR “Hypoglycaemic Agent” [tiab] OR “Antihyperglycaemic Agent” [tiab] OR “Hypoglycemic Agents” [tiab] OR “Antihyperglycemic Agents” [tiab] OR “Hypoglycaemic Agents” [tiab] OR “Antihyperglycaemic Agents” [tiab] OR "Dipeptidyl-Peptidase IV Inhibitors"[Mesh] OR “Dipeptidyl Peptidase IV” [tiab] OR “DPP-4” [tiab] OR “DPP 4” [tiab] OR “DPP-IV” [tiab] OR “DPP IV” [tiab] OR “Gliptins” [tiab] OR “Gliptin” [tiab] OR “Dipeptidyl Peptidase 4” [tiab] OR “Dipeptidyl-Peptidase IV Inhibitor” [tiab] OR “Dipeptidyl-Peptidase 4 Inhibitor” [tiab] OR “Dipeptidyl-Peptidase IV Inhibitors” [tiab] OR “Dipeptidyl-Peptidase 4 Inhibitors” [tiab] OR sitagliptin [tiab] OR vildagliptin [tiab] OR saxagliptin [tiab] OR alogliptin [tiab] OR linagliptin [tiab] OR gemigliptin [tiab] OR evogliptin [tiab] OR teneligliptin [tiab] OR "Adrenergic beta-Antagonists"[Mesh] OR alprenolol [Tiab] OR oxprenolol [Tiab] OR pindolol [Tiab] OR propranolol [Tiab] OR timolol [Tiab] OR sotalol [Tiab] OR nadolol [Tiab] OR mepindolol [Tiab] OR carteolol [Tiab] OR tertatolol [Tiab] OR bopindolol [Tiab] OR bupranolol [Tiab] OR penbutolol [Tiab] OR cloranolol [Tiab] OR practolol [Tiab] OR metoprolol [Tiab] OR atenolol [Tiab] OR acebutolol [Tiab] OR betaxolol [Tiab] OR bevantolol [Tiab] OR bisoprolol [Tiab] OR celiprolol [Tiab] OR esmolol [Tiab] OR epanolol [Tiab] OR s-atenolol [Tiab] OR nebivolol [Tiab] OR talinolol [Tiab] OR landiolol [Tiab] OR labetalol [Tiab] OR carvedilol [Tiab] OR “Adrenergic beta Antagonist” [Tiab] OR “Adrenergic beta-Antagonist” [Tiab] OR “Adrenergic beta Antagonists” [Tiab] OR “Adrenergic beta-Antagonists” [Tiab] OR “Beta blocker” [Tiab] OR “Beta-Blocker” [Tiab] OR “Beta blockers” [Tiab] OR “Beta-Blockers” [Tiab] OR "Angiotensin-Converting Enzyme Inhibitors"[Mesh] OR “Angiotensin Converting Enzyme” [Tiab] OR “Kininase II Inhibitor” [Tiab] OR “Kininase II Inhibitors” [Tiab] OR “Angiotensin-Converting Enzyme” [Tiab] OR “ACE Inhibitor” [Tiab] OR “ACE Inhibitors” [Tiab] OR “Angiotensin I-Converting Enzyme” [Tiab] OR “Angiotensin I Converting Enzyme” OR captopril [Tiab] OR enalapril [Tiab] OR lisinopril [Tiab] OR perindopril [Tiab] OR ramipril [Tiab] OR quinapril [Tiab] OR benazepril [Tiab] OR cilazapril [Tiab] OR fosinopril [Tiab] OR trandolapril [Tiab] OR spirapril [Tiab] OR delapril [Tiab] OR moexipril [Tiab] OR temocapril [Tiab] OR zofenopril [Tiab] OR imidapril [Tiab] OR Sacubitril [tiab] OR “neprilysin inhibitor” [tiab] OR “neprilysin inhibitors” [tiab] OR “NEP inhibitor”[tiab] OR “NEP inhibitors”[tiab]) AND ("Pneumonia, Aspiration"[Mesh] OR "Deglutition Disorders"[Mesh] OR dysphagia[Title/Abstract] OR "swallowing disorder"[Title/Abstract] OR "swallowing disorders"[Title/Abstract] OR "swallowing impairment"[Title/Abstract] OR "swallowing impairments"[Title/Abstract] OR "swallowing difficulty"[Title/Abstract] OR "swallowing difficulties"[Title/Abstract] OR "deglutition disorder"[Title/Abstract] OR "deglutition disorders"[Title/Abstract] OR "deglutition difficulty"[Title/Abstract] OR "deglutition difficulties"[Title/Abstract] OR "aspiration pneumonia"[Title/Abstract] OR pneumonia [Title/Abstract]))

**Scopus**

TITLE-ABS-KEY ( ( "antidiabetic*" OR "Hypoglycemic Agent*" OR "Antihyperglycemic Agent*" OR "Hypoglycaemic Agent*" OR "Antihyperglycaemic Agent*" OR "Dipeptidyl-Peptidase IV Inhibitor*" OR "Dipeptidyl Peptidase IV" OR "DPP-4" OR "DPP 4" OR "DPP-IV" OR "DPP IV" OR "Gliptin*" OR "Dipeptidyl Peptidase 4" OR "Dipeptidyl-Peptidase 4 Inhibitor" OR "sitagliptin" OR "vildagliptin" OR "saxagliptin" OR "alogliptin" OR "linagliptin" OR "gemigliptin" OR "evogliptin" OR "teneligliptin" OR "Adrenergic beta-Antagonist*" OR "alprenolol" OR "oxprenolol" OR "pindolol" OR "propranolol" OR "timolol" OR "sotalol" OR "nadolol" OR "mepindolol" OR "carteolol" OR "tertatolol" OR "bopindolol" OR "bupranolol" OR "penbutolol" OR "cloranolol" OR "practolol" OR "metoprolol" OR "atenolol" OR "acebutolol" OR "betaxolol" OR "bevantolol" OR "bisoprolol" OR "celiprolol" OR "esmolol" OR "epanolol" OR "s-atenolol" OR "nebivolol" OR "talinolol" OR "landiolol" OR "labetalol" OR "carvedilol" OR "Adrenergic beta Antagonist*" OR "Adrenergic beta-Antagonist*" OR "Beta blocker*" OR "Beta-Blocker*" OR "Angiotensin-Converting Enzyme Inhibitor*" OR "Angiotensin Converting Enzyme" OR "Kininase II Inhibitor*" OR "Angiotensin-Converting Enzyme" OR "ACE Inhibitor*" OR "Angiotensin I-Converting Enzyme" OR "Angiotensin I Converting Enzyme" OR "captopril" OR "enalapril" OR "lisinopril" OR "perindopril" OR "ramipril" OR "quinapril" OR "benazepril" OR "cilazapril" OR "fosinopril" OR "trandolapril" OR "spirapril" OR "delapril" OR "moexipril" OR "temocapril" OR "zofenopril" OR "imidapril" OR "Sacubitril" OR "neprilysin inhibitor*" OR "NEP inhibitor*" ) AND ( "Deglutition Disorder*" OR "dysphagia" OR "swallowing disorder*" OR "swallowing impairment*" OR "swallowing difficult*" OR "deglutition difficult*" OR "deglutition impairment*" OR "aspiration pneumonia" ) )

**Embase**

(('antidiabetic agent'/exp OR 'antidiabetes drug':ti,ab,kw OR 'antidiabetic':ti,ab,kw OR 'antidiabetic agent':ti,ab,kw OR 'antidiabetic agents':ti,ab,kw OR 'antidiabetic drug':ti,ab,kw OR 'antidiabetics':ti,ab,kw OR 'antidiabeticum':ti,ab,kw OR 'hypoglycaemic':ti,ab,kw OR 'hypoglycaemic agent':ti,ab,kw OR 'hypoglycaemic agents':ti,ab,kw OR 'hypoglycaemic compound':ti,ab,kw OR 'hypoglycaemic drug':ti,ab,kw OR 'hypoglycaemicising sulfonamide':ti,ab,kw OR 'hypoglycemic':ti,ab,kw OR 'hypoglycemic agent':ti,ab,kw OR 'hypoglycemic agents':ti,ab,kw OR 'hypoglycemic compound':ti,ab,kw OR 'hypoglycemic drug':ti,ab,kw OR 'hypoglycemicizing sulfonamide':ti,ab,kw OR 'hypoglycemizing agent':ti,ab,kw OR 'dipeptidyl peptidase iv inhibitor'/exp OR 'dpp 4 inhibitor':ti,ab,kw OR 'dpp iv inhibitor':ti,ab,kw OR 'dipeptidyl peptidase 4 inhibitor':ti,ab,kw OR 'dipeptidyl peptidase iv inhibitor':ti,ab,kw OR 'dipeptidyl peptidase iv inhibitors':ti,ab,kw OR 'dipeptidyl-peptidase iv inhibitors':ti,ab,kw OR 'dipeptidylpeptidase 4 inhibitor':ti,ab,kw OR 'dipeptidylpeptidase iv inhibitor':ti,ab,kw OR 'gliptin':ti,ab,kw OR 'gliptins':ti,ab,kw OR 'beta adrenergic receptor blocking agent'/exp OR 'adrenergic beta antagonists':ti,ab,kw OR 'adrenergic beta-antagonists':ti,ab,kw OR 'antiadrenergics, beta blocking':ti,ab,kw OR 'beta adrenergic antagonist':ti,ab,kw OR 'beta adrenergic blocker':ti,ab,kw OR 'beta adrenergic blockers':ti,ab,kw OR 'beta adrenergic blocking agent':ti,ab,kw OR 'beta adrenergic blocking drug':ti,ab,kw OR 'beta adrenergic receptor antagonist':ti,ab,kw OR 'beta adrenergic receptor blocker':ti,ab,kw OR 'beta adrenergic receptor blocking agent':ti,ab,kw OR 'beta adrenoceptor antagonist':ti,ab,kw OR 'beta adrenoceptor blocker':ti,ab,kw OR 'beta adrenoceptor blocking agent':ti,ab,kw OR 'beta adrenoceptor blocking drug':ti,ab,kw OR 'beta adrenolytic':ti,ab,kw OR 'beta adrenolytic agent':ti,ab,kw OR 'beta antagonist':ti,ab,kw OR 'beta antiadrenergic agent':ti,ab,kw OR 'beta blocker':ti,ab,kw OR 'beta blocking adrenergic agent':ti,ab,kw OR 'beta blocking agent':ti,ab,kw OR 'beta blocking drug':ti,ab,kw OR 'beta receptor adrenergic blocking agent':ti,ab,kw OR 'beta receptor blocker':ti,ab,kw OR 'beta receptor blocking agent':ti,ab,kw OR 'beta sympathicolytic agent':ti,ab,kw OR 'beta sympathicolytics':ti,ab,kw OR 'beta sympatholytic agent':ti,ab,kw OR 'betasympatholytic agent':ti,ab,kw OR 'dipeptidyl carboxypeptidase inhibitor'/exp OR 'ace inhibitor':ti,ab,kw OR 'angiotensin converting enzyme inhibiting agent':ti,ab,kw OR 'angiotensin converting enzyme inhibitor':ti,ab,kw OR 'angiotensin converting enzyme inhibitors':ti,ab,kw OR 'angiotensin i converting enzyme inhibitor':ti,ab,kw OR 'angiotensin-converting enzyme inhibitors':ti,ab,kw OR 'converting enzyme inhibitor':ti,ab,kw OR 'dipeptidyl carboxypeptidase i inhibitor':ti,ab,kw OR 'dipeptidyl carboxypeptidase inhibitor':ti,ab,kw OR 'kininase ii inhibitor':ti,ab,kw OR 'peptidyl dipeptidase inhibitor':ti,ab,kw OR 'peptidyldipeptide hydrolase inhibitor':ti,ab,kw OR 'enkephalinase inhibitor'/exp OR 'enkephalinase inhibitor':ti,ab,kw OR 'neprilysin inhibitor':ti,ab,kw OR 'neutral endopeptidase inhibitor':ti,ab,kw OR 'sitagliptin'/exp OR 'januvia' OR 'ristaben' OR 'sitagliptin' OR 'sitagliptin phosphate' OR 'sitagliptin phosphate monohydrate' OR 'sitagliptine' OR 'tesabel' OR 'tesavel' OR 'xelevia' OR 'vildagliptin'/exp OR 'galvus':ti,ab,kw OR 'jalra':ti,ab,kw OR 'vidagliptin':ti,ab,kw OR 'vildagliptin':ti,ab,kw OR 'xiliarx':ti,ab,kw OR 'saxagliptin'/exp OR 'onglyza':ti,ab,kw OR 'saxagliptin':ti,ab,kw OR 'saxagliptin hydrochloride':ti,ab,kw OR 'saxagliptin monohydrate':ti,ab,kw OR 'alogliptin'/exp OR 'alogliptin':ti,ab,kw OR 'alogliptin benzoate':ti,ab,kw OR 'nesina':ti,ab,kw OR 'vipidia':ti,ab,kw OR 'linagliptin'/exp OR 'linagliptin':ti,ab,kw OR 'ondero':ti,ab,kw OR 'tradjenta':ti,ab,kw OR 'trajenta':ti,ab,kw OR 'gemigliptin'/exp OR 'gemigliptin':ti,ab,kw OR 'evogliptin'/exp OR 'evogliptin':ti,ab,kw OR 'teneligliptin'/exp OR 'teneligliptin':ti,ab,kw OR 'alprenolol'/exp OR 'alfeprol':ti,ab,kw OR 'alloprenalol':ti,ab,kw OR 'alpheprol':ti,ab,kw OR 'alprendol':ti,ab,kw OR 'alprenol':ti,ab,kw OR 'alprenolol':ti,ab,kw OR 'alprenolol hydrochloride':ti,ab,kw OR 'alprenolol isomer':ti,ab,kw OR 'alprenololum':ti,ab,kw OR 'apliobal':ti,ab,kw OR 'aprenolol':ti,ab,kw OR 'aptia':ti,ab,kw OR 'aptin':ti,ab,kw OR 'aptin durules':ti,ab,kw OR 'aptine':ti,ab,kw OR 'aptol':ti,ab,kw OR 'aptol duriles':ti,ab,kw OR 'astra':ti,ab,kw OR 'betacard':ti,ab,kw OR 'betapin':ti,ab,kw OR 'gubernal':ti,ab,kw OR 'l alprenolol':ti,ab,kw OR 'levo dextro alprenolol':ti,ab,kw OR 'oxprenolol'/exp OR 'cordexol':ti,ab,kw OR 'coretal':ti,ab,kw OR 'd oxyprenolol':ti,ab,kw OR 'dextro oxyprenolol':ti,ab,kw OR 'l oxyprenolol':ti,ab,kw OR 'levo oxprenolol':ti,ab,kw OR 'levo oxyprenolol':ti,ab,kw OR 'oxprenol':ti,ab,kw OR 'oxprenolol':ti,ab,kw OR 'oxprenolol hydrochloride':ti,ab,kw OR 'oxtrenolol':ti,ab,kw OR 'oxyprenolol':ti,ab,kw OR 'oxyprenolol hydrochloride':ti,ab,kw OR 'slow trasicor':ti,ab,kw OR 'tevacor':ti,ab,kw OR 'trasacor':ti,ab,kw OR 'trasicor':ti,ab,kw OR 'trasicor retard':ti,ab,kw OR 'pindolol'/exp OR 'apo-pindol':ti,ab,kw OR 'apo-pindolol':ti,ab,kw OR 'barbloc':ti,ab,kw OR 'betapindol':ti,ab,kw OR 'blocklin':ti,ab,kw OR 'calvisken':ti,ab,kw OR 'carvisken':ti,ab,kw OR 'decreten':ti,ab,kw OR 'dranolis':ti,ab,kw OR 'durapindol':ti,ab,kw OR 'glauco visken':ti,ab,kw OR 'hexapindol':ti,ab,kw OR 'hydroxypropylaminopropoxyindol':ti,ab,kw OR 'nonspi':ti,ab,kw OR 'novo-pindol':ti,ab,kw OR 'pectobloc':ti,ab,kw OR 'pectoblock mite':ti,ab,kw OR 'pidol':ti,ab,kw OR 'pinbetol':ti,ab,kw OR 'pinden':ti,ab,kw OR 'pindol':ti,ab,kw OR 'pindolol':ti,ab,kw OR 'pindomex':ti,ab,kw OR 'pindoreal':ti,ab,kw OR 'pinloc':ti,ab,kw OR 'pinsken':ti,ab,kw OR 'prindolol':ti,ab,kw OR 'prinodolol':ti,ab,kw OR 'pyndale':ti,ab,kw OR 'treparasen':ti,ab,kw OR 'viskeen':ti,ab,kw OR 'viskeen retard':ti,ab,kw OR 'visken':ti,ab,kw OR 'visken mite':ti,ab,kw OR 'visken retard':ti,ab,kw OR 'viskene':ti,ab,kw OR 'vypen':ti,ab,kw OR 'propranolol'/exp OR 'acifol':ti,ab,kw OR 'adrexan':ti,ab,kw OR 'alperol':ti,ab,kw OR 'anaprilin':ti,ab,kw OR 'anapriline':ti,ab,kw OR 'anaprilinium':ti,ab,kw OR 'anapryline':ti,ab,kw OR 'angilol':ti,ab,kw OR 'angilol la':ti,ab,kw OR 'apo-propranolol':ti,ab,kw OR 'apsolol':ti,ab,kw OR 'arcablock':ti,ab,kw OR 'arcablock retard':ti,ab,kw OR 'artensol':ti,ab,kw OR 'authus':ti,ab,kw OR 'avlocardyl':ti,ab,kw OR 'avlocardyl retard':ti,ab,kw OR 'becardin':ti,ab,kw OR 'bedranol':ti,ab,kw OR 'beprane':ti,ab,kw OR 'bercolol':ti,ab,kw OR 'berkolol':ti,ab,kw OR 'beta neg':ti,ab,kw OR 'beta tablinen':ti,ab,kw OR 'beta tablinen retard':ti,ab,kw OR 'beta timelets':ti,ab,kw OR 'beta-timelets':ti,ab,kw OR 'betabloc':ti,ab,kw OR 'betadipresan':ti,ab,kw OR 'betaneg':ti,ab,kw OR 'betaprol':ti,ab,kw OR 'betares':ti,ab,kw OR 'betaryl':ti,ab,kw OR 'blocard':ti,ab,kw OR 'blocaryl':ti,ab,kw OR 'cardinol':ti,ab,kw OR 'cardinol la':ti,ab,kw OR 'ciplar':ti,ab,kw OR 'corbeta':ti,ab,kw OR 'deralin':ti,ab,kw OR 'dextrolevo propranolol':ti,ab,kw OR 'dibudinate':ti,ab,kw OR 'dideral':ti,ab,kw OR 'dl propanolol hydrochloride':ti,ab,kw OR 'dl propranolol':ti,ab,kw OR 'dociton':ti,ab,kw OR 'dociton retard':ti,ab,kw OR 'docitone':ti,ab,kw OR 'durabeton':ti,ab,kw OR 'duranol':ti,ab,kw OR 'efektolol':ti,ab,kw OR 'efektolol retard':ti,ab,kw OR 'elbrol':ti,ab,kw OR 'emforal':ti,ab,kw OR 'farmadral':ti,ab,kw OR 'farprolol':ti,ab,kw OR 'frekven':ti,ab,kw OR 'frina':ti,ab,kw OR 'hemangeol':ti,ab,kw OR 'hemangiol':ti,ab,kw OR 'hopranolol':ti,ab,kw OR 'ikopal':ti,ab,kw OR 'impral':ti,ab,kw OR 'inderal':ti,ab,kw OR 'inderal la':ti,ab,kw OR 'inderal retard':ti,ab,kw OR 'inderalici':ti,ab,kw OR 'inderex':ti,ab,kw OR 'indicardin':ti,ab,kw OR 'indobloc':ti,ab,kw OR 'innopran':ti,ab,kw OR 'innopran xl':ti,ab,kw OR 'inpanol':ti,ab,kw OR 'ipran':ti,ab,kw OR 'l propranolol':ti,ab,kw OR 'lederpronol':ti,ab,kw OR 'levo propranolol':ti,ab,kw OR 'levopropranolol':ti,ab,kw OR 'napriline':ti,ab,kw OR 'noloten':ti,ab,kw OR 'obsidan':ti,ab,kw OR 'obsin':ti,ab,kw OR 'obzidan':ti,ab,kw OR 'oposim':ti,ab,kw OR 'phanerol':ti,ab,kw OR 'prandol':ti,ab,kw OR 'prano puren':ti,ab,kw OR 'pranopuren':ti,ab,kw OR 'prestoral':ti,ab,kw OR 'prolol':ti,ab,kw OR 'prolol plus':ti,ab,kw OR 'pronovan':ti,ab,kw OR 'propabloc':ti,ab,kw OR 'propal':ti,ab,kw OR 'propalong':ti,ab,kw OR 'propanolol hydrochloride':ti,ab,kw OR 'propayerst':ti,ab,kw OR 'propercuten':ti,ab,kw OR 'prophylux':ti,ab,kw OR 'propra ratiopharm':ti,ab,kw OR 'propral':ti,ab,kw OR 'propranolol':ti,ab,kw OR 'propranolol hydrochloride':ti,ab,kw OR 'propranolol hydrochloride intensol':ti,ab,kw OR 'propranolol isomer':ti,ab,kw OR 'propranur':ti,ab,kw OR 'proprasylyt':ti,ab,kw OR 'proprasylyte':ti,ab,kw OR 'reducor':ti,ab,kw OR 'sagittol':ti,ab,kw OR 'slow deralin':ti,ab,kw OR 'stapranolol':ti,ab,kw OR 'sumial':ti,ab,kw OR 'tenomal':ti,ab,kw OR 'tensiflex':ti,ab,kw OR 'waucoton':ti,ab,kw OR 'timolol'/exp OR 'apo timolol':ti,ab,kw OR 'apo-timolol':ti,ab,kw OR 'apotimolol':ti,ab,kw OR 'timolo':ti,ab,kw OR 'timolol':ti,ab,kw OR 'timolol hemihydrate':ti,ab,kw OR 'titol':ti,ab,kw OR 'sotalol'/exp OR 'alosot':ti,ab,kw OR 'beta-cardone':ti,ab,kw OR 'betacardone':ti,ab,kw OR 'betades':ti,ab,kw OR 'betapace':ti,ab,kw OR 'betapace af':ti,ab,kw OR 'bonpro':ti,ab,kw OR 'corsotalol':ti,ab,kw OR 'd sotalol':ti,ab,kw OR 'darob':ti,ab,kw OR 'dexsotalol':ti,ab,kw OR 'dexsotalol hydrochloride':ti,ab,kw OR 'dextro sotalol':ti,ab,kw OR 'dextrosotalol':ti,ab,kw OR 'favorex':ti,ab,kw OR 'gilucor':ti,ab,kw OR 'hipecor':ti,ab,kw OR 'isotalol':ti,ab,kw OR 'jutalex':ti,ab,kw OR 'l sotalol':ti,ab,kw OR 'levo sotalol':ti,ab,kw OR 'levosotalol':ti,ab,kw OR 'rentibloc':ti,ab,kw OR 'rotalol':ti,ab,kw OR 'satalol':ti,ab,kw OR 'satolol':ti,ab,kw OR 'so aqueous':ti,ab,kw OR 'solavert':ti,ab,kw OR 'sorine':ti,ab,kw OR 'sota saar':ti,ab,kw OR 'sotab':ti,ab,kw OR 'sotabeta':ti,ab,kw OR 'sotacol':ti,ab,kw OR 'sotacor':ti,ab,kw OR 'sotahexal':ti,ab,kw OR 'sotalex':ti,ab,kw OR 'sotalol':ti,ab,kw OR 'sotalol hydrochloride':ti,ab,kw OR 'sotaper':ti,ab,kw OR 'sotapor':ti,ab,kw OR 'sotastad':ti,ab,kw OR 'sotylize':ti,ab,kw OR 'tachytalol':ti,ab,kw OR 'nadolol'/exp OR 'apo-nadol' OR 'apo-nadolol' OR 'betadol' OR 'corgard' OR 'farmagard' OR 'nadic' OR 'nadolol' OR 'solgol' OR 'solgol mite' OR 'mepindolol'/exp OR 'betagon':ti,ab,kw OR 'caridian':ti,ab,kw OR 'corindolan':ti,ab,kw OR 'mepindolol':ti,ab,kw OR 'mepindolol sulfate':ti,ab,kw OR 'mepindolol sulphate':ti,ab,kw OR 'pindolol, methyl':ti,ab,kw OR 'carteolol'/exp OR 'arteolol':ti,ab,kw OR 'arteoptic':ti,ab,kw OR 'arteoptik':ti,ab,kw OR 'caltamol':ti,ab,kw OR 'calte':ti,ab,kw OR 'carbonolol':ti,ab,kw OR 'carteabak':ti,ab,kw OR 'carteol':ti,ab,kw OR 'carteol lp':ti,ab,kw OR 'carteolol':ti,ab,kw OR 'carteolol hydrochloride':ti,ab,kw OR 'cartrol':ti,ab,kw OR 'elebloc':ti,ab,kw OR 'endak':ti,ab,kw OR 'endak mite':ti,ab,kw OR 'glauteolol':ti,ab,kw OR 'karol':ti,ab,kw OR 'karteol':ti,ab,kw OR 'mikelan':ti,ab,kw OR 'tertatolol'/exp OR 'artex':ti,ab,kw OR 'prenalex':ti,ab,kw OR 'tertatolol':ti,ab,kw OR 'tertatolol hydrochloride':ti,ab,kw OR 'bopindolol'/exp OR 'bopindolol' OR 'sandonorm' OR 'wandonorm' OR 'bupranolol'/exp OR 'betadrenol':ti,ab,kw OR 'bupranolol':ti,ab,kw OR 'bupranolol hydrochloride':ti,ab,kw OR 'ophtorenin':ti,ab,kw OR 'panimit':ti,ab,kw OR 'penbutolol'/exp OR 'betapresin':ti,ab,kw OR 'betapressin':ti,ab,kw OR 'blocotin':ti,ab,kw OR 'levatol':ti,ab,kw OR 'paginol':ti,ab,kw OR 'penbutalol':ti,ab,kw OR 'penbutolol':ti,ab,kw OR 'penbutolol sulfate':ti,ab,kw OR 'penbutolol sulphate':ti,ab,kw OR 'cloranolol'/exp OR 'cloranolol':ti,ab,kw OR 'tobanum':ti,ab,kw OR 'practolol'/exp OR 'dalzic':ti,ab,kw OR 'dextro levo practolol':ti,ab,kw OR 'dl practolol':ti,ab,kw OR 'eraldin':ti,ab,kw OR 'eraldin inj.':ti,ab,kw OR 'practalol':ti,ab,kw OR 'practolol':ti,ab,kw OR 'practolole':ti,ab,kw OR 'praktolol':ti,ab,kw OR 'praktololu':ti,ab,kw OR 'proctalol':ti,ab,kw OR 'teranol':ti,ab,kw OR 'metoprolol'/exp OR 'beloc duriles':ti,ab,kw OR 'belok zok':ti,ab,kw OR 'betaloc':ti,ab,kw OR 'metoprolol':ti,ab,kw OR 'metoprolol durules':ti,ab,kw OR 'metoprolol oros':ti,ab,kw OR 'metropolol':ti,ab,kw OR 'atenolol'/exp OR 'ablok':ti,ab,kw OR 'adoll':ti,ab,kw OR 'alonet':ti,ab,kw OR 'altol':ti,ab,kw OR 'anolene':ti,ab,kw OR 'anolpin':ti,ab,kw OR 'anselol':ti,ab,kw OR 'apo-atenolol':ti,ab,kw OR 'arandin':ti,ab,kw OR 'asten':ti,ab,kw OR 'atarox':ti,ab,kw OR 'atcardil':ti,ab,kw OR 'atecard':ti,ab,kw OR 'atehexal':ti,ab,kw OR 'atelol':ti,ab,kw OR 'atenblock':ti,ab,kw OR 'atendol':ti,ab,kw OR 'atenet':ti,ab,kw OR 'ateni':ti,ab,kw OR 'atenil':ti,ab,kw OR 'ateno':ti,ab,kw OR 'atenogamma':ti,ab,kw OR 'atenol':ti,ab,kw OR 'atenolol':ti,ab,kw OR 'atereal':ti,ab,kw OR 'aterol':ti,ab,kw OR 'atestad':ti,ab,kw OR 'atinol':ti,ab,kw OR 'atolmin':ti,ab,kw OR 'b-vasc':ti,ab,kw OR 'betablok':ti,ab,kw OR 'betacar':ti,ab,kw OR 'betarol':ti,ab,kw OR 'betatop ge':ti,ab,kw OR 'beten':ti,ab,kw OR 'bloket':ti,ab,kw OR 'blokium':ti,ab,kw OR 'blotex':ti,ab,kw OR 'cardioten':ti,ab,kw OR 'catenol':ti,ab,kw OR 'coratol':ti,ab,kw OR 'corotenol':ti,ab,kw OR 'durabeta':ti,ab,kw OR 'esatenolol':ti,ab,kw OR 'evitocor':ti,ab,kw OR 'farnormin':ti,ab,kw OR 'felo-bits':ti,ab,kw OR 'hypernol':ti,ab,kw OR 'hypoten':ti,ab,kw OR 'internolol':ti,ab,kw OR 'lo-ten':ti,ab,kw OR 'loten':ti,ab,kw OR 'lotenal':ti,ab,kw OR 'martenol':ti,ab,kw OR 'mirobect':ti,ab,kw OR 'myocord':ti,ab,kw OR 'neotenol':ti,ab,kw OR 'nolol':ti,ab,kw OR 'normalol':ti,ab,kw OR 'normaten':ti,ab,kw OR 'normiten':ti,ab,kw OR 'nortelol':ti,ab,kw OR 'noten':ti,ab,kw OR 'oraday':ti,ab,kw OR 'ormidol':ti,ab,kw OR 'paesumex':ti,ab,kw OR 'plenacor':ti,ab,kw OR 'preloc':ti,ab,kw OR 'premorine':ti,ab,kw OR 'prenolol':ti,ab,kw OR 'prenormine':ti,ab,kw OR 'ranlol':ti,ab,kw OR 'rozamin':ti,ab,kw OR 'serten':ti,ab,kw OR 'stermin':ti,ab,kw OR 'temoret':ti,ab,kw OR 'tenblok':ti,ab,kw OR 'tenidon':ti,ab,kw OR 'tenoblock':ti,ab,kw OR 'tenocor':ti,ab,kw OR 'tenol':ti,ab,kw OR 'tenolin':ti,ab,kw OR 'tenolol':ti,ab,kw OR 'tenopress':ti,ab,kw OR 'tenoprin':ti,ab,kw OR 'tenormin':ti,ab,kw OR 'tenormin mite':ti,ab,kw OR 'tenormine':ti,ab,kw OR 'tenostat':ti,ab,kw OR 'tensig':ti,ab,kw OR 'tensinor':ti,ab,kw OR 'ternolol':ti,ab,kw OR 'therabloc':ti,ab,kw OR 'tredol':ti,ab,kw OR 'vascoten':ti,ab,kw OR 'velorin':ti,ab,kw OR 'vericordin':ti,ab,kw OR 'wesipin':ti,ab,kw OR 'acebutolol'/exp OR 'acebutolol':ti,ab,kw OR 'acebutolol hydrochloride':ti,ab,kw OR 'acecor':ti,ab,kw OR 'diasectral':ti,ab,kw OR 'espesil':ti,ab,kw OR 'flebutol':ti,ab,kw OR 'grifobutol':ti,ab,kw OR 'monitan':ti,ab,kw OR 'neptall':ti,ab,kw OR 'prent':ti,ab,kw OR 'rhotral':ti,ab,kw OR 'sectral':ti,ab,kw OR 'sectral ge':ti,ab,kw OR 'sectral lp':ti,ab,kw OR 'betaxolol'/exp OR 'alcon betoptic':ti,ab,kw OR 'betac':ti,ab,kw OR 'betarun':ti,ab,kw OR 'betasel':ti,ab,kw OR 'betaxolol':ti,ab,kw OR 'betaxolol hydrochloride':ti,ab,kw OR 'betaxon':ti,ab,kw OR 'betoptic':ti,ab,kw OR 'betoptic s':ti,ab,kw OR 'betoptima':ti,ab,kw OR 'betoquin':ti,ab,kw OR 'kerlon':ti,ab,kw OR 'kerlone':ti,ab,kw OR 'kerlong':ti,ab,kw OR 'levobetaxolol':ti,ab,kw OR 'levobetaxolol hydrochloride':ti,ab,kw OR 'lokren':ti,ab,kw OR 'optibet':ti,ab,kw OR 'optipress':ti,ab,kw OR 'tonobexol':ti,ab,kw OR 'bevantolol'/exp OR 'benatol':ti,ab,kw OR 'bevantolol':ti,ab,kw OR 'bevantolol hydrochloride':ti,ab,kw OR 'bevantololum':ti,ab,kw OR 'calvan':ti,ab,kw OR 'ranestol':ti,ab,kw OR 'vantol':ti,ab,kw OR 'bisoprolol'/exp OR 'bisoprolol':ti,ab,kw OR 'celiprolol'/exp OR 'abecor':ti,ab,kw OR 'celectol':ti,ab,kw OR 'celiprolol':ti,ab,kw OR 'celiprolol hydrochloride':ti,ab,kw OR 'dilanorm':ti,ab,kw OR 'selecor':ti,ab,kw OR 'selectol':ti,ab,kw OR 'esmolol'/exp OR 'brevibloc':ti,ab,kw OR 'brevibloc double strength':ti,ab,kw OR 'esmolol':ti,ab,kw OR 'esmolol hydrochloride':ti,ab,kw OR 'miniblock':ti,ab,kw OR 'epanolol'/exp OR 'epanolol':ti,ab,kw OR 'visacor (epanolol)':ti,ab,kw OR 'nebivolol'/exp OR 'bivolet':ti,ab,kw OR 'bystolic':ti,ab,kw OR 'dexnebivolol':ti,ab,kw OR 'hypoloc':ti,ab,kw OR 'insucor':ti,ab,kw OR 'levonebivolol':ti,ab,kw OR 'lobivon':ti,ab,kw OR 'narbivolol':ti,ab,kw OR 'nebilet':ti,ab,kw OR 'nebiloc':ti,ab,kw OR 'nebilox':ti,ab,kw OR 'nebivolol':ti,ab,kw OR 'nebivolol hydrochloride':ti,ab,kw OR 'nobistar':ti,ab,kw OR 'nobiten':ti,ab,kw OR 'nomexor':ti,ab,kw OR 'silostar':ti,ab,kw OR 'temerit':ti,ab,kw OR 'talinolol'/exp OR 'cordanum':ti,ab,kw OR 'talinolol':ti,ab,kw OR 'landiolol'/exp OR 'landiobloc':ti,ab,kw OR 'landiolol':ti,ab,kw OR 'landiolol hydrochloride':ti,ab,kw OR 'onoact':ti,ab,kw OR 'rapibloc':ti,ab,kw OR 'raploc':ti,ab,kw OR 'runrapiq':ti,ab,kw OR 'labetalol'/exp OR 'abetol':ti,ab,kw OR 'albetol':ti,ab,kw OR 'amipress':ti,ab,kw OR 'biascor':ti,ab,kw OR 'hybloc':ti,ab,kw OR 'ibidomide':ti,ab,kw OR 'ipolab':ti,ab,kw OR 'labelol':ti,ab,kw OR 'labesine':ti,ab,kw OR 'labetalol':ti,ab,kw OR 'labetalol hydrochloride':ti,ab,kw OR 'labetolol':ti,ab,kw OR 'lamitol':ti,ab,kw OR 'liondox':ti,ab,kw OR 'normodyne':ti,ab,kw OR 'presdate':ti,ab,kw OR 'presolol':ti,ab,kw OR 'pressalolo':ti,ab,kw OR 'salmagne':ti,ab,kw OR 'trandate':ti,ab,kw OR 'carvedilol'/exp OR 'cardiol (carvedilol)':ti,ab,kw OR 'cardivas':ti,ab,kw OR 'carvedilol':ti,ab,kw OR 'carvedilol phosphate':ti,ab,kw OR 'carvedlol':ti,ab,kw OR 'carvipress':ti,ab,kw OR 'carvrol':ti,ab,kw OR 'coreg':ti,ab,kw OR 'coreg cr':ti,ab,kw OR 'dilatrend':ti,ab,kw OR 'dilbloc':ti,ab,kw OR 'dimitone':ti,ab,kw OR 'eucardic':ti,ab,kw OR 'kredex':ti,ab,kw OR 'querto':ti,ab,kw OR 'v-bloc':ti,ab,kw OR 'enalapril'/exp OR 'enalapril':ti,ab,kw OR 'lisinopril'/exp OR 'acerbon':ti,ab,kw OR 'alapril':ti,ab,kw OR 'alfaken':ti,ab,kw OR 'carace':ti,ab,kw OR 'cipril':ti,ab,kw OR 'coric':ti,ab,kw OR 'dapril':ti,ab,kw OR 'fibsol':ti,ab,kw OR 'inopril':ti,ab,kw OR 'linopril':ti,ab,kw OR 'linvas':ti,ab,kw OR 'lipril':ti,ab,kw OR 'lisi abz':ti,ab,kw OR 'lisibeta':ti,ab,kw OR 'lisigamma':ti,ab,kw OR 'lisihexal':ti,ab,kw OR 'lisinopril':ti,ab,kw OR 'lisinopril dihydrate':ti,ab,kw OR 'lisipril':ti,ab,kw OR 'lisodur':ti,ab,kw OR 'lisopress':ti,ab,kw OR 'lisopril':ti,ab,kw OR 'lisoril':ti,ab,kw OR 'lispril':ti,ab,kw OR 'listril':ti,ab,kw OR 'lysinopril':ti,ab,kw OR 'noperten':ti,ab,kw OR 'novatec':ti,ab,kw OR 'presiten':ti,ab,kw OR 'prinil':ti,ab,kw OR 'prinivil':ti,ab,kw OR 'qbrelis':ti,ab,kw OR 'sinopril':ti,ab,kw OR 'tensopril':ti,ab,kw OR 'tensyn':ti,ab,kw OR 'vivatec':ti,ab,kw OR 'zestomax':ti,ab,kw OR 'zestril':ti,ab,kw OR 'perindopril'/exp OR 'armix arginin':ti,ab,kw OR 'bioprexanil':ti,ab,kw OR 'coverex':ti,ab,kw OR 'coverex forte':ti,ab,kw OR 'coversoral':ti,ab,kw OR 'coversum':ti,ab,kw OR 'coversum arginine':ti,ab,kw OR 'coversyl':ti,ab,kw OR 'coversyl arginine':ti,ab,kw OR 'mariper':ti,ab,kw OR 'perindopril':ti,ab,kw OR 'perindopril arginine':ti,ab,kw OR 'perindoprilarginin':ti,ab,kw OR 'perineva':ti,ab,kw OR 'prestarium':ti,ab,kw OR 'prestarium arginine':ti,ab,kw OR 'prexanil':ti,ab,kw OR 'procaptan':ti,ab,kw OR 'ramipril'/exp OR 'acovil':ti,ab,kw OR 'altace':ti,ab,kw OR 'carasel':ti,ab,kw OR 'cardace':ti,ab,kw OR 'corpril':ti,ab,kw OR 'delix':ti,ab,kw OR 'hypren':ti,ab,kw OR 'hytren':ti,ab,kw OR 'lostapres':ti,ab,kw OR 'pramace':ti,ab,kw OR 'ramace':ti,ab,kw OR 'ramilich':ti,ab,kw OR 'ramipril':ti,ab,kw OR 'triatec':ti,ab,kw OR 'tritace':ti,ab,kw OR 'unipril':ti,ab,kw OR 'vesdil':ti,ab,kw OR 'vivace (ramipril)':ti,ab,kw OR 'quinapril'/exp OR 'accupril':ti,ab,kw OR 'accuprin':ti,ab,kw OR 'accupro':ti,ab,kw OR 'accupron':ti,ab,kw OR 'acequin':ti,ab,kw OR 'acuitel':ti,ab,kw OR 'acuprel':ti,ab,kw OR 'acupril':ti,ab,kw OR 'asig':ti,ab,kw OR 'conan':ti,ab,kw OR 'ectren':ti,ab,kw OR 'korec':ti,ab,kw OR 'quinalapril':ti,ab,kw OR 'quinapril':ti,ab,kw OR 'quinapril hcl':ti,ab,kw OR 'quinapril hydrochloride':ti,ab,kw OR 'quinaten':ti,ab,kw OR 'quinazil':ti,ab,kw OR 'quinhexal':ti,ab,kw OR 'quinipril':ti,ab,kw OR 'benazepril'/exp OR 'benace':ti,ab,kw OR 'benazepril':ti,ab,kw OR 'benazepril hydrochloride':ti,ab,kw OR 'boncordin':ti,ab,kw OR 'briem':ti,ab,kw OR 'brien':ti,ab,kw OR 'cibace':ti,ab,kw OR 'cibacen':ti,ab,kw OR 'cibacen cor':ti,ab,kw OR 'cibacene':ti,ab,kw OR 'fortekor':ti,ab,kw OR 'lotensin':ti,ab,kw OR 'tenkuoren':ti,ab,kw OR 'zinadril':ti,ab,kw OR 'cilazapril'/exp OR 'cilazapril':ti,ab,kw OR 'cilazipril':ti,ab,kw OR 'cliazapril':ti,ab,kw OR 'dynorm':ti,ab,kw OR 'inhibace':ti,ab,kw OR 'inibace':ti,ab,kw OR 'initiss':ti,ab,kw OR 'inocar':ti,ab,kw OR 'justor':ti,ab,kw OR 'vascace':ti,ab,kw OR 'vascase':ti,ab,kw OR 'fosinopril'/exp OR 'acenor-m':ti,ab,kw OR 'bpnorm':ti,ab,kw OR 'dynacil':ti,ab,kw OR 'eliten':ti,ab,kw OR 'fosenopril':ti,ab,kw OR 'fosenopril sodium':ti,ab,kw OR 'fosinil':ti,ab,kw OR 'fosinonorm':ti,ab,kw OR 'fosinopril':ti,ab,kw OR 'fosinopril sodium':ti,ab,kw OR 'fosinorm':ti,ab,kw OR 'fosipres':ti,ab,kw OR 'fositen':ti,ab,kw OR 'fositens':ti,ab,kw OR 'fovas':ti,ab,kw OR 'fozitec':ti,ab,kw OR 'monopril':ti,ab,kw OR 'newace':ti,ab,kw OR 'sapril':ti,ab,kw OR 'staril':ti,ab,kw OR 'tensogard':ti,ab,kw OR 'vasopril':ti,ab,kw OR 'trandolapril'/exp OR 'gopten':ti,ab,kw OR 'mavik':ti,ab,kw OR 'odace':ti,ab,kw OR 'odric':ti,ab,kw OR 'odrik':ti,ab,kw OR 'trandolapril':ti,ab,kw OR 'udrik':ti,ab,kw OR 'spirapril'/exp OR 'equaten':ti,ab,kw OR 'quadropil':ti,ab,kw OR 'quadropril':ti,ab,kw OR 'renormax':ti,ab,kw OR 'renpress':ti,ab,kw OR 'sandopril':ti,ab,kw OR 'spirapril':ti,ab,kw OR 'spirapril hydrochloride':ti,ab,kw OR 'wandopres':ti,ab,kw OR 'delapril'/exp OR 'adecut':ti,ab,kw OR 'alindapril':ti,ab,kw OR 'delapril':ti,ab,kw OR 'delapril hydrochloride':ti,ab,kw OR 'moexipril'/exp OR 'fampress':ti,ab,kw OR 'femipres':ti,ab,kw OR 'fempres':ti,ab,kw OR 'fempress':ti,ab,kw OR 'frempress':ti,ab,kw OR 'moex':ti,ab,kw OR 'moexipril':ti,ab,kw OR 'moexipril hydrochloride':ti,ab,kw OR 'perdix':ti,ab,kw OR 'primoxil':ti,ab,kw OR 'tensotec':ti,ab,kw OR 'univasc':ti,ab,kw OR 'univase':ti,ab,kw OR 'temocapril'/exp OR 'temocapril':ti,ab,kw OR 'temocapril hydrochloride':ti,ab,kw OR 'acecol':ti,ab,kw OR 'zofenopril'/exp OR 'bifril':ti,ab,kw OR 'zofenil':ti,ab,kw OR 'zofenopril':ti,ab,kw OR 'zofenopril calcium':ti,ab,kw OR 'zofenoprilum':ti,ab,kw OR 'zopranol':ti,ab,kw OR 'imidapril'/exp OR 'imidapril':ti,ab,kw OR 'imidapril hydrochloride':ti,ab,kw OR 'novarok':ti,ab,kw OR 'tanapril':ti,ab,kw OR 'tanatril':ti,ab,kw OR 'sacubitril plus valsartan'/exp OR 'entresto':ti,ab,kw OR 'neparvis':ti,ab,kw OR 'sacubitril plus valsartan':ti,ab,kw OR 'sacubitril/valsartan':ti,ab,kw OR 'valsartan plus sacubitril':ti,ab,kw OR 'valsartan/sacubitril':ti,ab,kw) AND ('dysphagia'/mj OR 'deglutition difficulty' OR 'deglutition disorder' OR 'deglutition disorders' OR 'difficult deglutition' OR 'difficulty in swallowing' OR 'difficulty swallowing' OR 'dysphagia' OR 'dysphagias' OR 'swallowing difficult' OR 'swallowing difficultness' OR 'swallowing difficulty' OR 'swallowing disorder' OR 'aspiration pneumonia'/mj OR 'aspiration pneumonia' OR 'aspiration pneumonitis' OR 'deglutition pneumonia' OR 'pneumonia, aspiration'))

**CINAHL**

1. ( (MH "Hypoglycemic Agents+") OR (MH "Dipeptidyl Peptidase 4 Inhibitors+") OR (MH "Adrenergic Beta-Antagonists+") OR (MH "Angiotensin-Converting Enzyme Inhibitors+") ) OR ( “antidiabetic” OR “Hypoglycemic Agent” OR “Antihyperglycemic Agent” OR “Hypoglycaemic Agent” OR “Antihyperglycaemic Agent” OR “Hypoglycemic Agents” OR “Antihyperglycemic Agents” OR “Hypoglycaemic Agents” OR “Antihyperglycaemic Agents” OR “Dipeptidyl Peptidase IV” OR “DPP-4” OR “DPP 4” OR “DPP-IV” OR “DPP IV” OR “Gliptins” OR “Gliptin” OR “Dipeptidyl Peptidase 4” OR “Dipeptidyl-Peptidase IV Inhibitor” OR “Dipeptidyl-Peptidase 4 Inhibitor” OR “Dipeptidyl-Peptidase IV Inhibitors” OR “Dipeptidyl-Peptidase 4 Inhibitors” OR sitagliptin OR vildagliptin OR saxagliptin OR alogliptin OR linagliptin OR gemigliptin OR evogliptin OR teneligliptin OR alprenolol OR oxprenolol OR pindolol OR propranolol OR timolol OR sotalol OR nadolol OR mepindolol OR carteolol OR tertatolol OR bopindolol OR bupranolol OR penbutolol OR cloranolol OR practolol OR metoprolol OR atenolol OR acebutolol OR betaxolol OR bevantolol OR bisoprolol OR celiprolol OR esmolol OR epanolol OR s-atenolol OR nebivolol OR talinolol OR landiolol OR labetalol OR carvedilol OR “Adrenergic beta Antagonist” OR “Adrenergic beta-Antagonist” OR “Adrenergic beta Antagonists” OR “Adrenergic beta-Antagonists” OR “Beta blocker” OR “Beta-Blocker” OR “Beta blockers” OR “Beta-Blockers” OR “Angiotensin Converting Enzyme” OR “Kininase II Inhibitor” OR “Kininase II Inhibitors” OR “Angiotensin-Converting Enzyme” OR “ACE Inhibitor” OR “ACE Inhibitors” OR “Angiotensin I-Converting Enzyme” OR “Angiotensin I Converting Enzyme” OR captopril OR enalapril OR lisinopril OR perindopril OR ramipril OR quinapril OR benazepril OR cilazapril OR fosinopril OR trandolapril OR spirapril OR delapril OR moexipril OR temocapril OR zofenopril OR imidapril OR Sacubitril OR “neprilysin inhibitor” OR “neprilysin inhibitors” OR “NEP inhibitor” OR “NEP inhibitors” )
2. ( (MH "Pneumonia, Aspiration") OR (MH "Deglutition Disorders") ) OR AB ( ( dysphagia OR 'swallowing disorder' OR 'swallowing disorders' OR 'swallowing impairment' OR 'swallowing impairments' OR 'swallowing difficulty' OR 'swallowing difficulties' OR 'deglutition disorder' OR 'deglutition disorders' OR 'deglutition difficulty' OR 'deglutition difficulties' OR 'deglutition impairment' OR 'deglutition impairments' OR 'aspiration pneumonia' OR pneumonia ) )
3. #1 AND #2

**Cochrane**

**1.** MeSH descriptor: [Deglutition Disorders] explode all trees

**2.** MeSH descriptor: [Pneumonia, Aspiration] this term only

**3.** (dysphagia OR 'swallowing disorder' OR 'swallowing disorders' OR 'swallowing impairment' OR 'swallowing impairments' OR 'swallowing difficulty' OR 'swallowing difficulties' OR 'deglutition disorder' OR 'deglutition disorders' OR 'deglutition difficulty' OR 'deglutition difficulties' OR 'deglutition impairment' OR 'deglutition impairments' OR 'aspiration pneumonia' OR pneumonia):ti,ab,kw

**4.** #1 OR #2 OR #3

**5.** MeSH descriptor: [Hypoglycemic Agents] explode all trees

**6.** MeSH descriptor: [Dipeptidyl-Peptidase IV Inhibitors] explode all trees

**7.** MeSH descriptor: [Adrenergic beta-Antagonists] explode all trees

**8.** MeSH descriptor: [Angiotensin-Converting Enzyme Inhibitors] explode all trees

**9.** (“antidiabetic” OR “Hypoglycemic Agent” OR “Antihyperglycemic Agent” OR “Hypoglycaemic Agent” OR “Antihyperglycaemic Agent” OR “Hypoglycemic Agents” OR “Antihyperglycemic Agents” OR “Hypoglycaemic Agents” OR “Antihyperglycaemic Agents” OR “Dipeptidyl Peptidase IV” OR “DPP-4” OR “DPP 4” OR “DPP-IV” OR “DPP IV” OR “Gliptins” OR “Gliptin” OR “Dipeptidyl Peptidase 4” OR “Dipeptidyl-Peptidase IV Inhibitor” OR “Dipeptidyl-Peptidase 4 Inhibitor” OR “Dipeptidyl-Peptidase IV Inhibitors” OR “Dipeptidyl-Peptidase 4 Inhibitors” OR sitagliptin OR vildagliptin OR saxagliptin OR alogliptin OR linagliptin OR gemigliptin OR evogliptin OR teneligliptin OR alprenolol OR oxprenolol OR pindolol OR propranolol OR timolol OR sotalol OR nadolol OR mepindolol OR carteolol OR tertatolol OR bopindolol OR bupranolol OR penbutolol OR cloranolol OR practolol OR metoprolol OR atenolol OR acebutolol OR betaxolol OR bevantolol OR bisoprolol OR celiprolol OR esmolol OR epanolol OR s-atenolol OR nebivolol OR talinolol OR landiolol OR labetalol OR carvedilol OR “Adrenergic beta Antagonist” OR “Adrenergic beta-Antagonist” OR “Adrenergic beta Antagonists” OR “Adrenergic beta-Antagonists” OR “Beta blocker” OR “Beta-Blocker” OR “Beta blockers” OR “Beta-Blockers” OR “Angiotensin Converting Enzyme” OR “Kininase II Inhibitor” OR “Kininase II Inhibitors” OR “Angiotensin-Converting Enzyme” OR “ACE Inhibitor” OR “ACE Inhibitors” OR “Angiotensin I-Converting Enzyme” OR “Angiotensin I Converting Enzyme” OR captopril OR enalapril OR lisinopril OR perindopril OR ramipril OR quinapril OR benazepril OR cilazapril OR fosinopril OR trandolapril OR spirapril OR delapril OR moexipril OR temocapril OR zofenopril OR imidapril OR Sacubitril OR “neprilysin inhibitor” OR “neprilysin inhibitors” OR “NEP inhibitor” OR “NEP inhibitors”):ti,ab,kw

**10.** #5 OR #6 OR #7 OR #8 OR #9

**11.** #4 AND #10
