# Supplementary material for: On the potential of drug repurposing in dysphagia treatment: New insights from a real-world pharmacovigilance study and a systematic review
Source: Front Pharmacol. 2023 Mar 3;14:1057301. doi: 10.3389/fphar.2023.1057301 (PMC10022593; doi:10.3389/fphar.2023.1057301)
Supplement: Supplementary file 2 [file Table2.DOCX]

Supplementary Material

**Supplementary Material S2. Concomitant drugs known to increase the risk of dysphagia (alphabetical order)**

ACEPROMAZINE

ACETOPHENAZINE

AGOMELATINE

ALAPROCLATE

AMINEPTINE

AMISULPRIDE

AMITRIPTYLINE

AMOXAPINE

ARIPIPRAZOLE

ASENAPINE

BENPERIDOL

BIFEMELANE

BOTULINUM

BREXPIPRAZOLE

BROMOCRIPTINE

BROMPERIDOL

BUPROPION

BUTAPERAZINE

BUTRIPTYLINE

CARIPRAZINE

CERITINIB

CERIVASTATIN

CHLORPHENIRAMINE

CHLORPROETHAZINE

CHLORPROMAZINE

CHLORPROTHIXENE

CITALOPRAM

CLOMIPRAMINE

CLOPENTHIXOL

CLOTIAPINE

CLOZAPINE

CYAMEMAZINE

DANTROLENE

DESIPRAMINE

DESVENLAFAXINE

DIBENZEPIN

DICLOFENAC

DIMETACRINE

DIPHENHYDRAMINE

DIXYRAZINE

DOCETAXEL

DONEPEZIL

DOSULEPIN

DOXEPIN

DROPERIDOL

DULOXETINE

ENTRECTINIB

EPOETIN

ESCITALOPRAM

ESKETAMINE

ETIDRONATE

ETOPERIDONE

EVEROLIMUS

FENOPROFEN

FLUANISONE

FLUOXETINE

FLUPENTIXOL

FLUPHENAZINE

FLUSPIRILENE

FLUVASTATIN

FLUVOXAMINE

GALANTAMINE

GEPIRONE

GINKGO

HALOPERIDOL

HOMATROPINE

HYDROXOCOBALAMIN

HYOSCYAMINE

HYPERICI

IBANDRONATE

IBUPROFEN

ILOPERIDONE

IMIPRAMINE

INDOMETHACIN

IPIDACRINE

IPRINDOLE

IPROCLOZIDE

IPRONIAZIDE

ISOCARBOXAZID

KETOPROFEN

KETOROLAC

LEVOMEPROMAZINE

LEVOSULPIRIDE

LITHIUM

LOFEPRAMINE

LOVASTATIN

LOXAPINE

LUMATEPERONE

LURASIDONE

MAPROTILINE

MECLOFENAMATE

MEDIFOXAMINE

MEFENAMIC

MELITRACEN

MELPERONE

MEMANTINE

MESORIDAZINE

MIANSERIN

MILNACIPRAN

MINAPRINE

MIRTAZAPINE

MOCLOBEMIDE

MOLINDONE

MOPERONE

MOSAPRAMINE

MYCOPHENOLATE

NAPROXEN

NEFAZODONE

NIACIN

NIALAMIDE

NIVOLUMAB

NOMIFENSINE

NORTRIPTYLINE

OLANZAPINE

OPIPRAMOL

OXAFLOZANE

OXAPROZIN

OXITRIPTAN

OXYCODONE

OXYPERTINE

PALIPERIDONE

PAROXETINE

PEMBROLIZUMAB

PENFLURIDOL

PERAZINE

PERICIAZINE

PERPHENAZINE

PHENELZINE

PIMAVANSERIN

PIMOZIDE

PIPAMPERONE

PIPOTIAZINE

PIROXICAM

PIVAGABINE

PORFIMER

PROCHLORPERAZINE

PROMAZINE

PROTHIPENDYL

PROTRIPTYLINE

PSEUDOEPHEDRINE

QUETIAPINE

QUINUPRAMINE

REBOXETINE

REMOXIPRIDE

RISEDRONATE

RISPERIDONE

RIVASTIGMINE

ROFECOXIB

SCOPOLAMINE

SERTINDOLE

SERTRALINE

SIMVASTATIN

SULINDAC

SULPIRIDE

SULTOPRIDE

TACRINE

THIOPROPAZATE

THIOPROPERAZINE

THIORIDAZINE

THIOTHIXENE

TIANEPTINE

TIAPRIDE

TILUDRONATE

TIOTIXENE

TOLMETIN

TOLOXATONE

TRANYLCYPROMINE

TRAZODONE

TRIFLUOPERAZINE

TRIFLUOPERAZINE

TRIFLUPERIDOL

TRIFLUPROMAZINE

TRIMIPRAMINE

TRYPTOPHAN

VALDECOXIB

VENLAFAXINE

VERALIPRIDE

VILAZODONE

VILOXAZINE

VORTIOXETINE

ZIMELDINE

ZIPRASIDONE

ZOTEPINE

ZUCLOPENTHIXOL
