# Supplementary material for: On the potential of drug repurposing in dysphagia treatment: New insights from a real-world pharmacovigilance study and a systematic review
Source: Front Pharmacol. 2023 Mar 3;14:1057301. doi: 10.3389/fphar.2023.1057301 (PMC10022593; doi:10.3389/fphar.2023.1057301)
Supplement: Supplementary file 3 [file Table3.DOCX]

Supplementary Material

**Supplementary material**

**Figure S3.** Risk of bias of individual RCTs using the RoB2
