# Supplementary material for: On the potential of drug repurposing in dysphagia treatment: New insights from a real-world pharmacovigilance study and a systematic review
Source: Front Pharmacol. 2023 Mar 3;14:1057301. doi: 10.3389/fphar.2023.1057301 (PMC10022593; doi:10.3389/fphar.2023.1057301)
Supplement: Supplementary file 4 [file Table4.DOCX]

Supplementary Material

**Table S4.** Methodological quality of nRCTs, single-arm CTs, and observational studies using the ROBINS-I assessment tool

| First Author year | Risk of bias pre-intervention and at-intervention domains | | | Risk of bias post-intervention domains | | | | Overall  Assessment  of bias |
| --- | --- | --- | --- | --- | --- | --- | --- | --- |
|  | **Bias due**  **to Confounding** | **Bias in selection of participants into the study** | **Bias in classification of interventions** | **Bias due to deviations from intended intervention** | **Bias due to missing data** | **Bias in measurement of outcomes** | **Bias in selection of the reported result** |  |
| Arai 1998a | NO INFORMATION | LOW | SERIUOS | NO INFORMATION | NO INFORMATION | MODERATE | LOW | NO INFORMATION |
| Arai 1998 b | NO INFORMATION | LOW | SERIOUS | NO INFORMATION | NO INFORMATION | MODERATE | LOW | NO INFORMATION |
| Arai 2001 | LOW | LOW | SERIOUS | NO INFORMATION | NO INFORMATION | MODERATE | LOW | NO INFORMATION |
| Arai 2005 | LOW | SERIOUS | MODERATE | NO INFORMATION | NO INFORMATION | MODERATE | LOW | NO INFORMATION |
| Bosch 2012 | SERIOUS | SERIOUS | SERIOUS | NO INFORMATION | LOW | MODERATE | LOW | CRITICAL |
| Fernandes 2021 | LOW | MODERATE | SERIOUS | NO INFORMATION | LOW | MODERATE | LOW | CRITICAL |
| Kumazawa 2019 | MODERATE | LOW | LOW | NO INFORMATION | LOW | MODERATE | LOW | MODERATE |
| Liu 2012 | MODERATE | MODERATE | LOW | NO INFORMATION | LOW | MODERATE | LOW | MODERATE |
| Marciniak 2008 | CRITICAL | MODERATE | CRITICAL | NO INFORMATION | LOW | MODERATE | LOW | CRITICAL |
| Matsumoto 2012 | NO INFORMATION | LOW | NO INFORMATION | NO INFORMATION | NO INFORMATION | SERIOUS | MODERATE | NO INFORMATION |
| Miarons 2016 | MODERATE | MODERATE | SERIOUS | NO INFORMATION | LOW | MODERATE | LOW | SERIOUS |
| Miarons 2018 | LOW | SERIOUS | MODERATE | CRITICAL | LOW | MODERATE | LOW | CRITICAL |
| Noguchi 2020 | SERIOUS | SERIOUS | SERIOUS | NO INFORMATION | NO INFORMATION | SERIOUS | LOW | CRITICAL |
| Sato 2013 | NO INFORMATION | NO INFORMATION | NO INFORMATION | NO INFORMATION | NO INFORMATION | MODERATE | MODERATE | NO INFORMATION |
| Shimizu 2008 | SERIOUS | LOW | LOW | NO INFORMATION | LOW | LOW | MODERATE | SERIOUS |

LEGEND

| NO INFORMATION | CRITICAL | SERIOUS | MODERATE | LOW |
| --- | --- | --- | --- | --- |

+ Risk of bias - Risk of bias
